# Cell‐Free DNA‐Based Theranostics for Inflammatory Disorders

**DOI:** 10.1002/advs.202520383

**Published:** 2026-01-20

**Authors:** Jiatong Li, Yumei Wang, Xiaoxi Fan, Miao Xu, Jiawen Li, Wenfei Tan, Jianxiang Zhang, Heran Li

**Affiliations:** ^1^ School of Pharmacy China Medical University Shenyang China; ^2^ Department of Pharmaceutics College of Pharmacy Army Medical University Chongqing China; ^3^ Department of Thoracic Surgery The First Hospital of China Medical University Shenyang China; ^4^ Department of Anesthesiology The First Hospital of China Medical University Shenyang China

**Keywords:** cell‐free DNA, cGAS‐STING pathway, inflammation, inflammatory disorders, toll‐like receptors

## Abstract

Inflammatory disorders are characterized by immune‐mediated inflammatory cascades that can affect multiple organs. Cell‐free DNA (cfDNA) is not only a biomarker for various inflammatory diseases, but also a driver in innate immune responses, offering emerging diagnostic and therapeutic possibilities for inflammatory diseases. This review begins by examining immune signatures in inflammation, with particular focus on the vicious cycle between cfDNA and inflammatory responses. We then discuss cfDNA detection strategies and their clinical applications as disease biomarkers. Crucially, we highlight design principles and formulation strategies for cfDNA‐based interventions for inflammation regulation, considering physical, biochemical, and immunological properties of cfDNA. These approaches encompass advanced nanotechnologies such as drug loading, targeted delivery, inflammation‐responsive release, and microenvironment reprogramming. Subsequently, we examine cfDNA intervention strategies for precision treatment of inflammatory diseases, including inflammatory bowel diseases, rheumatoid arthritis, sepsis, periodontitis, and psoriasis. Finally, we present key insights and future perspectives, as well as discuss translational challenges and clinical considerations, thereby paving the way for innovative approaches to inflammation modulation and disease management.

## Graphical abstract

Summary on the dual potential of cfDNA as biomarkers and therapeutic targets for inflammatory disorders. Figure was created with BioRender.com.

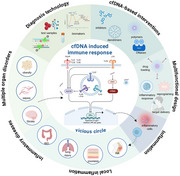

## 1 Introduction

Inflammation, defined as the defensive physiological response of the immune system to internal and external stimuli (e.g. infection, trauma, or harmful substances), is the body's natural response to maintain tissue homeostasis, usually modulating innate immune cells and manifesting symptoms such as redness [1], swelling [2], heat [3], pain [4], etc. Appropriate inflammation is crucial in maintaining the general homeostasis, but an abnormal, excessive, or inadequate inflammatory response may lead to tissue damage, cytokine storm, and organ dysfunction, which is one of the major contributors to numerous acute or chronic inflammatory disorders [5, 6, 7, 8, 9, 10, 11, 12]. Many inflammatory diseases, such as sepsis [13], inflammatory bowel diseases (IBD) [14], rheumatoid arthritis (RA) [15], stroke [16], periodontal disease [17], psoriasis and atherosclerosis [18], despite the different lesion locations and clinical manifestations, fall into this category, all of which seriously affect patients' life and bring significant economic burdens for public health systems worldwide [19]. Clinically, anti‐inflammatory, immunomodulatory, and antibacterial drugs, including non‐steroidal anti‐inflammatory drugs (NSAIDs) [20, 21, 22], glucocorticoids [23], immunosuppressive agents [24], biological agents [25], and antibiotics [26] are routinely used for the etiological and symptomatic treatments of inflammatory diseases [27, 28]. Although these medications partly relieve the clinical symptoms, they are often ineffective in addressing the underlying problem of inflammation as a consequence of their poor pharmacokinetics and lack of specificity and targeting [29, 30]. In addition, long‐term use of these treatments may lead to drug resistance and side effects, including gastrointestinal discomfort, liver and kidney injury, osteoporosis, and opportunistic infections [31, 32, 33]. Accordingly, the search for novel therapeutic strategies has become a clinical imperative.

Over the past decades, studies have confirmed the overlapping immunopathogenesis and a close association among inflammatory disorders [34]. Typically, pathogen‐associated molecular patterns (PAMPs) and damage‐associated molecular patterns (DAMPs) [35, 36], which are recognized by pattern‐recognition receptors (PRRs) to mediate intrinsic immunity, elicit the inflammatory responses and activate downstream signaling pathways [37, 38]. During inflammation, damaged cells undergoing necrosis or apoptosis release a large amount of DNA into the circulation, which is denoted as cell‐free DNA (cfDNA) [39], and is increasingly regarded as a biomarker to reflect the degree of tissue injury. Additionally, cfDNA has been demonstrated to be an important pathogenic signal, such as PAMPs/DAMPs, to induce further persistent inflammatory responses to cause cell death and tissue damage, thus creating a vicious cycle [40, 41].

**FIGURE 1:**
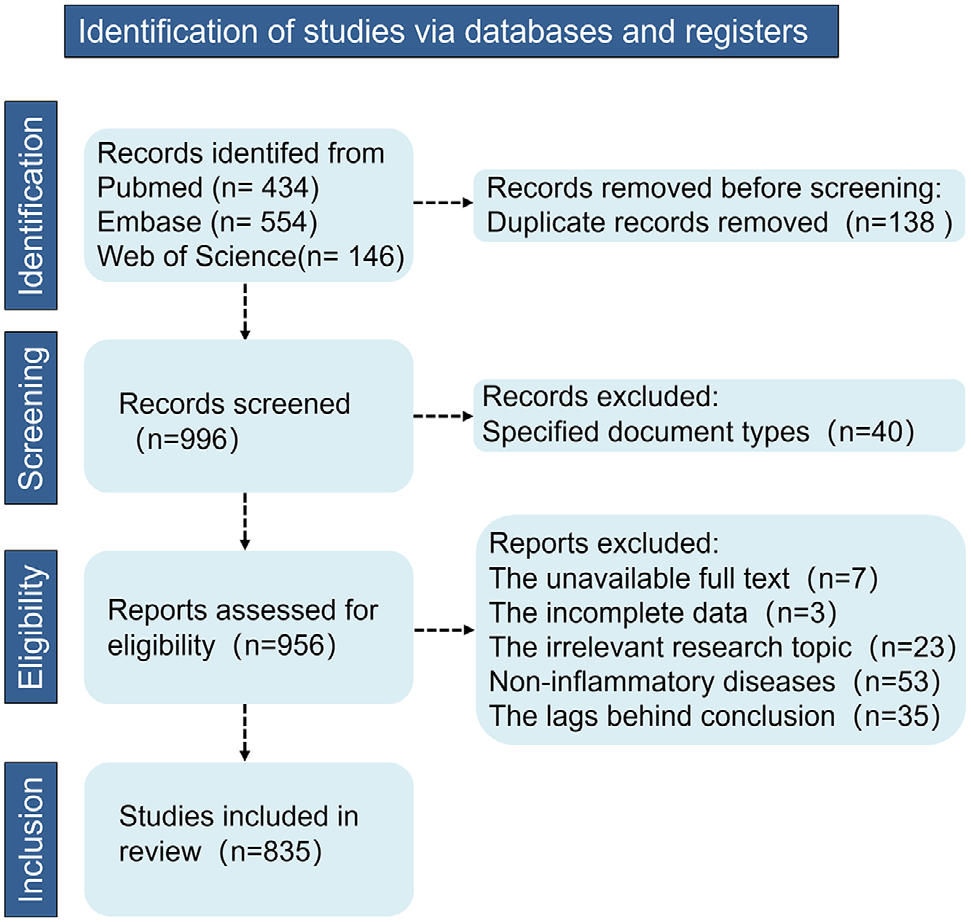
PRISMA flow diagram. The PRISMA flow diagram illustrates the systematic screening of study identification, screening, eligibility assessment, and final inclusion, which reflects the comprehensive search across three databases, detailing exclusions at each stage and the final inclusion of 835 studies for further review.

Here, a PRISMA flow diagram is developed to outline the selection process from databases (e.g., PubMed, Embase, and Web of Science), excluding the duplicate, non‐relevant, outdated, inaccessible, or incomplete literature, to facilitate subsequent literature review (Figure 1). In this article, we discuss the vicious circle between cfDNA and inflammatory responses, review the detection strategies and clinical applications of cfDNA as a biomarker of various diseases, and highlight the design principles and formulation strategies of cfDNA‐based interventions for inflammation regulation. Blocking the generation of cfDNA, degradation or scavenging the excess cfDNA, and inhibiting the inflammatory cascades with the assistance of small molecules, polymers, enzymes, and nucleic acid drugs are considered as emerging therapeutic strategies for inflammatory diseases by modulating the body's innate immune response [42]. Moreover, nanomedicines with a large spectrum of composition, morphology, surface chemistry, and surface modification offer multi‐functional designs such as drug loading, targeted delivery, inflammation‐responsive release, and microenvironment reprogramming, thereby providing precise manipulation and effective treatment on inflammatory diseases. By unlocking the dual potential of cfDNA, revolutionizing developments in the diagnosis and treatment of inflammatory disorders, as well as more precise immune manipulation, can be translated from bench to bedside.

## 2 The Vicious Circle Between cfDNA and Inflammatory Diseases

### 2.1 Concept and Development of cfDNA

**FIGURE 2:**
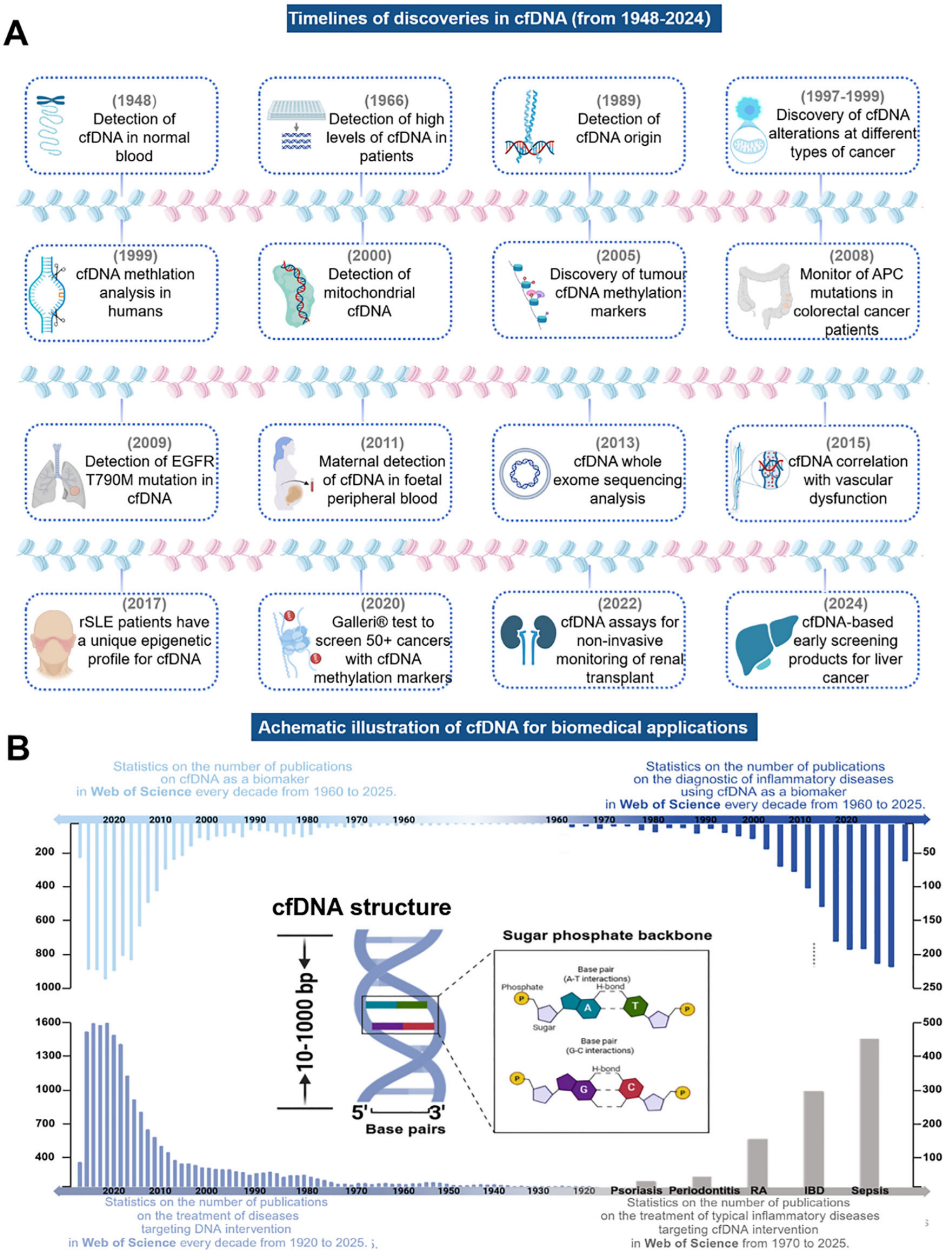
Development of cfDNA. (A) Timeline diagram illustrating the first identification of cfDNA, its evolution as a potential biomarker, and milestone discoveries in biomedical applications across multiple diseases. (B) Statistical analysis of the existing literature on the cfDNA biomedical applications in Web of Science. Figure was created with http://www.BioRender.com.

**FIGURE 3:**
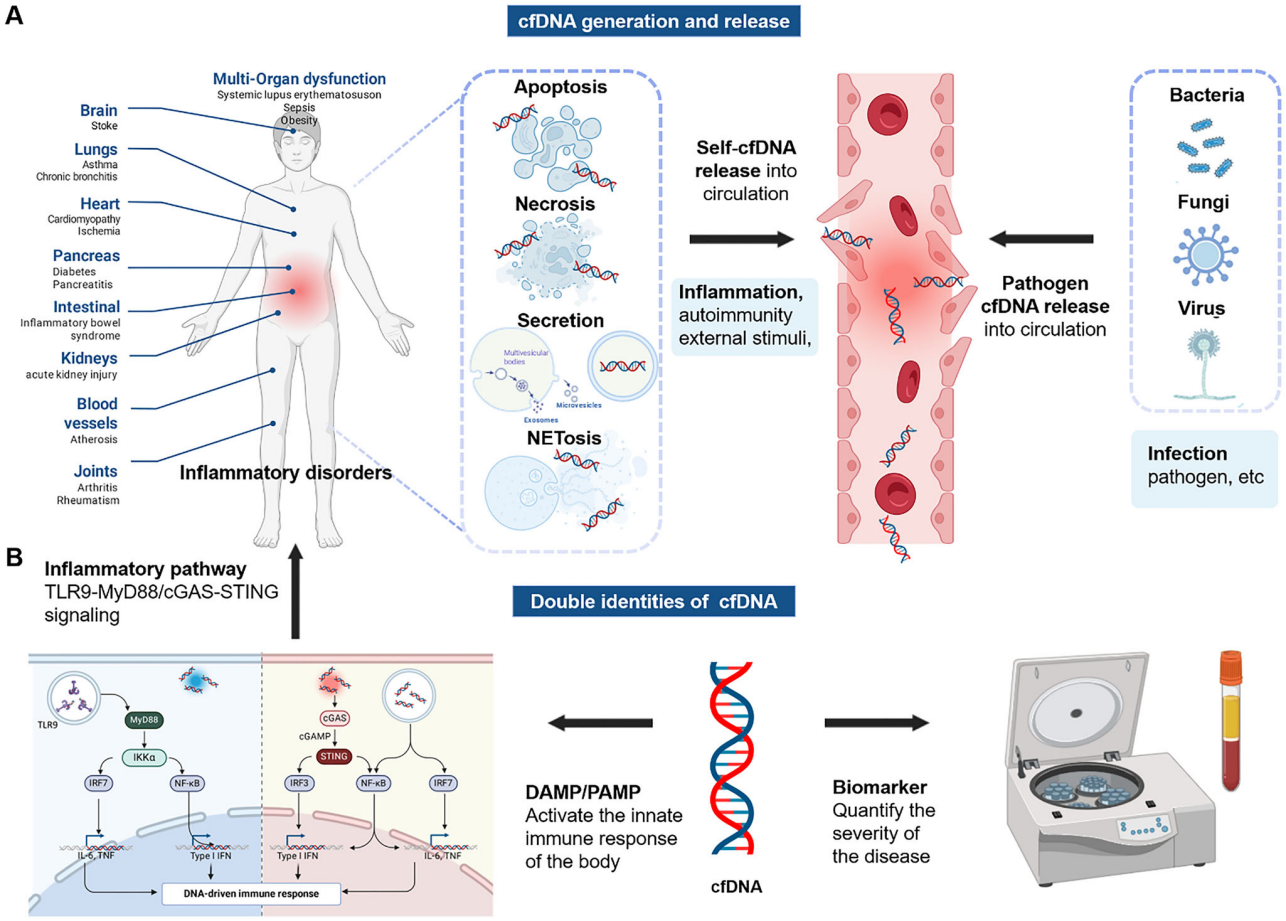
cfDNA generation and its dual roles in inflammatory diseases. (A) Depending on its origin, cfDNA can be categorized into self‐cfDNA and pathogen cfDNA. The former is primarily generated through cellular apoptosis, necrosis, active secretion, and NETosis. (B) Schematic diagram illustrating cfDNA's dual roles as both a biomarker and a disease driver. Figure was created with http://www.BioRender.com.

**FIGURE 4:**
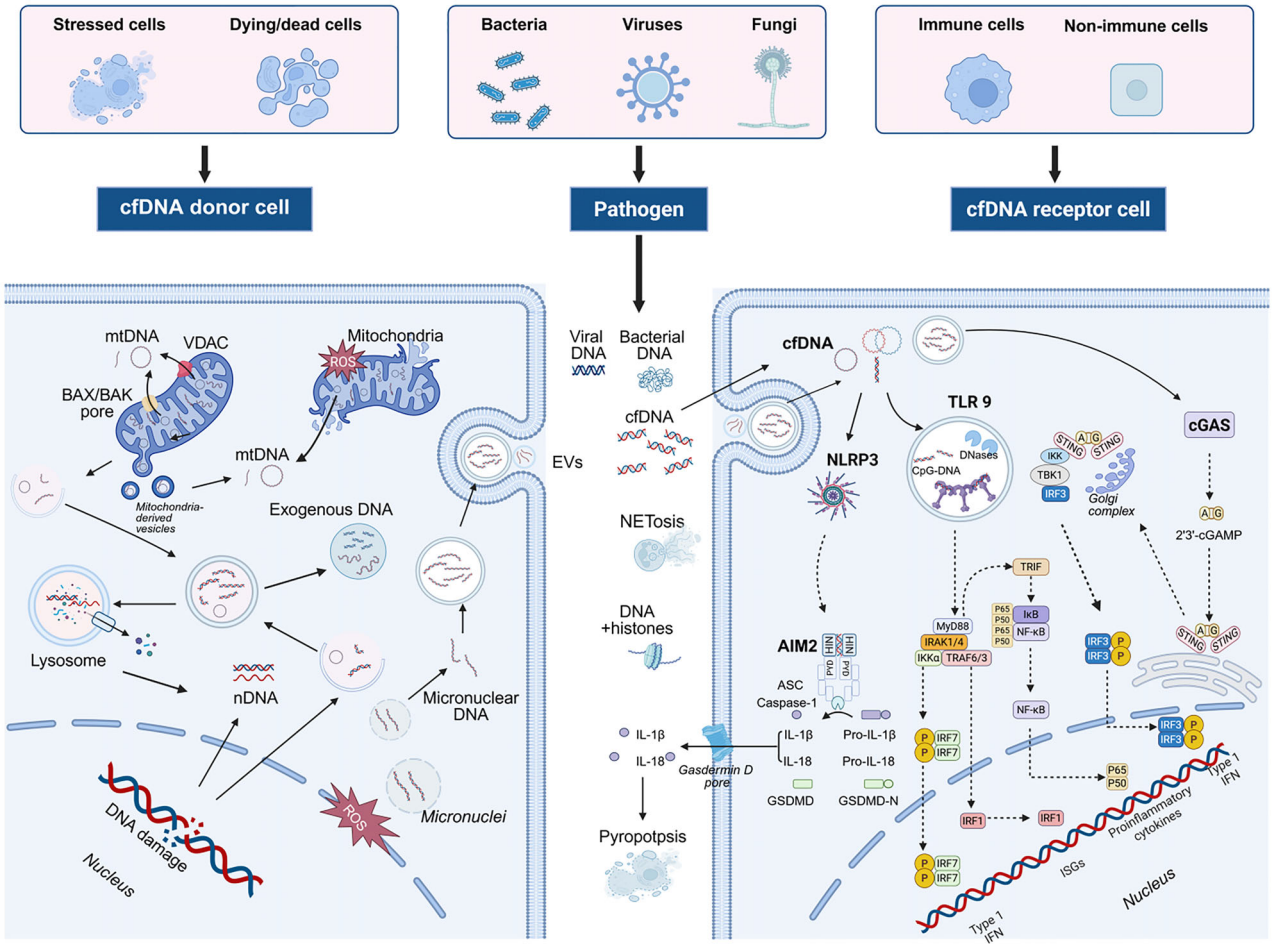
Origins, transport pathways, and immune activation mechanisms of cfDNA. cfDNA primarily originates from damaged pathogens, cellular stress, cellular injury, cell death, and active release processes, such as NETosis and extracellular vesicles. During this process, cell‐free DNA is able to bind to proteins such as histones and HMGB1. Additionally, subcellular components, including mitochondria, nucleus, micronuclei, and lysosomes, can be impaired, leading to the release of nucleic acid. TLR9 specifically recognizes CpG‐DNA and recruits MyD88 to induce IRF7‐mediated IFN‐I release, IRF1‐mediated ISGs expression, and NF‐κB‐mediated pro‐inflammatory cytokine release. cfDNA also activates cGAS, catalyzing the synthesis of cGAMP, which then activates STING, ultimately promoting IRF3‐mediated type I IFN release and NF‐κB‐mediated release of cytokines, chemokines, and enzymes. Moreover, cfDNA, especially mtDNA, bound to AIM2 drives NLRP3 inflammasome assembly with ASC and caspase‐1, initiating the production of IL‐1β and IL‐18 as well as pyroptosis. Meanwhile, EndoG damages mitochondria and triggers the release of mtDNA and ROS, activating the corresponding signaling pathways. Figure created with BioRender.com. Abbreviations: EndoG, endonuclease G; HIN, hematopoietic interferon‐inducible nuclear; ISGs, interferon‐stimulated genes; IκB, inhibitor of nuclear factor kappa B; IKK, inhibitor of kappa B kinase; IRF, interferon regulatory factor; MIP, macrophage inflammatory protein; iNOS, inducible nitric oxide synthase; PYD, pyrin domain; ROS, reactive oxygen species. Figure was created with http://www.BioRender.com.

cfDNA denotes extracellular DNA fragments present in biological fluids (such as blood, cerebrospinal fluid, and urine), and exists in single‐stranded, double‐stranded, or circular forms, primarily originating from damaged pathogens and biological processes of the organism, such as cell apoptosis, necrosis, and active secretion [43, 44]. cfDNA fragments typically ranges from 10 to 100 base pairs (bp) in length, though some may exceed 1000 bp [45, 46]. Extracellular cfDNA was first detected in human blood in 1948 [47, 48]. Subsequent studies have shown its potential association with the pathogenesis of inflammatory diseases such as cancer and rheumatic systemic lupus erythematosus (rSLE), establishing its dual role as both a biomarker and a potential pathogenic driver [49] (Figures 2 and 3). With the development of analytical biology and diagnostic technology, cfDNA carries genetic and epigenetic information reflecting its origin, making it a critical biomarker for non‐invasive diagnostics and disease monitoring [50, 51]. Based on its origins, (such as cellular apoptosis, necrosis, active secretion, and neutrophil extracellular trap formation [NETosis] or pathogens) (Figures 3 and 4) and biological characteristics, cfDNA can be classified into several major categories, including pathogen‐derived unmethylated cytosine‐guanine dinucleotides (CpG DNA), bacterial DNA (bDNA), mitochondrial cfDNA (mtDNA), nuclear DNA (nDNA), circulating tumor DNA (ctDNA), neutrophil extracellular capture networks (NETs), fetal DNA in maternal circulation and exosome‐associated DNA actively secreted by viable cells [52, 53]. In healthy individuals, the concentration of cfDNA is very low, and can be cleared by deoxyribonuclease I (DNase I) to maintain the dynamic balance [54]. Significant elevation in cfDNA level is observed in individuals with benign lesion, infection, inflammatory disease, tissue trauma, etc. [55, 56, 57]. Thus, cfDNA natively serves as a clinical biomarker, offering critical insights for the diagnosis, prognosis, and therapeutic management of various diseases.

### 2.2 Relationship Between Inflammation and cfDNA

The management of inflammatory diseases continues to pose substantial clinical challenges, primarily due to their intricate and multifactorial pathogenesis [58, 59, 60, 61, 62, 63, 64]. Emerging evidence has implicated cfDNA as a key contributor to the progression of inflammatory diseases [65, 66, 67, 68, 69, 70]. Mechanistically, cfDNA released from damaged or dead cells activates DNA sensors that exacerbate the pathogenesis of inflammatory diseases [69]. Notably, cfDNA orchestrates both innate and adaptive immune responses through multiple mechanisms [71] (Figure 4). Immune complexes formed by cfDNA with auto‐antibodies (e.g., anti‐citrullinated protein antibodies [72]) or DNA‐binding proteins (e.g., HMGB1 [73], LL37 [74]) are internalized by antigen‐presenting cells, triggering Toll‐like receptor (TLR)‐dependent secretion of pro‐inflammatory cytokines. Typically, when cfDNA enters the endosomes of immune cells, such as macrophages or dendritic cells, the host immune response is initiated by recognizing it as DAMPs, causing significant illness [75, 76, 77, 78]. This cascade also amplifies inflammatory responses and activates T/B lymphocytes, thereby potentiating systemic immunity and playing a crucial role in cellular aging and natural aging processes.

For example, cfDNA can be recognized and bound by Toll‐like receptor‐9 (TLR9), which then interacts with the downstream TLRs adaptor myeloid differential protein‐88 (MyD88) junction protein and triggers a series of downstream signaling pathways to predominantly culminate in type I interferon (IFN‐I) [79, 80, 81, 82, 83]. TLR9 is a DNA sensor that responds to the DNase II cleavage products of pathogens‐derived and/or self‐DNA (especially mtDNA) containing unmethylated CpG dinucleotides. The interaction between TLR9 and MyD88 first contributes to the activation of nuclear factor Kappa‐B (NF‐κB), where the recruited tumor necrosis factor receptor‐associated factor 6 (TRAF6) activates transforming growth factor β (TGF‐β) associated kinase 1 (TAK1), which phosphorylates inhibitor of NF‐κB (IKK) complex and leads to NF‐κB activation and proinflammatory cytokine release. MyD88 promotes nuclear translocation of interferon regulatory factor 1 (IRF1) to induce expression of IFN stimulates [84, 85, 86, 87]. Second, TLR9‐MyD88 signaling also causes a complex formed with tumor necrosis factor receptor‐associated factor (TRAF) [88], interleukin‐1 (IL‐1) receptor‐associated kinase (IRAK) 1 and IRAK4 [89], which activates interferon regulatory factor (IRF) to translocate into the nucleus and induce type I IFN production [90].

Additionally, intracellular cfDNA stimulates the cyclic GMP‐AMP synthase (cGAS)‐stimulator of interferon genes (STING) pathway, initiating IFN‐I responses. The cGAS‐STING signaling pathway is considered the only non‐redundant pathway for sensing cytosolic double‐stranded DNA (dsDNA). cGAS‐STING pathway is always activated by cytosolic DNA, including both pathogen‐derived and self‐DNA [91, 92, 93, 94, 95]. After a conformation change, the DNA‐bound cGAS forms a catalytic pocket to synthesize cyclic guanosine monophosphate‐adenosine monophosphate (cGAMP) from guanosine triphosphate (GTP) and adenosine triphosphate (ATP). STING, as the sensor protein of cGAMP, plays a central role in the downstream signaling [96, 97, 98, 99, 100]. When bound to cGAMP, STING undergoes oligomerization and migrates from the endoplasmic reticulum membrane to the Golgi apparatus via COPII vesicles, recruiting TANK‐binding kinase 1 (TBK1) and inducing the gene expression of Type I IFN response, interferon‐stimulated genes (ISGs), and several other inflammatory mediators, pro‐apoptotic genes, and chemokines to initiate downstream signal transduction. On the other hand, STING can induce NF‐κB activation and cause transcription of inflammatory cytokines via TBK1, mediating autophagy and programmed cell death. In addition, nuclear domain protein absents in melanoma 2 (AIM2) is also a dsDNA detector, which can bind dsDNA and drive inflammasome assembly with acid‐sensing channel (ASC) and caspase‐1, triggering the release of interleukin‐1β (IL‐1β) and interleukin‐18 (IL‐18) as well as pyroptosis [101, 102, 103, 104, 105].

Furthermore, cfDNA has complex interactions with inflammatory mediators, such as reactive oxygen species (ROS), and further drives the onset and progression of inflammatory diseases. In an inflammatory state, excess ROS with strong oxidation properties, attacks proteins and nucleotides, destroys the DNA structure, and causes cell rupture or apoptosis, thus leading to the release of cfDNA [106, 107, 108, 109, 110, 111, 112]. In turn, cfDNA activates relevant signaling pathways by binding to the receptors on the surface of immune cells and modulating intracellular ROS production [113, 114, 115, 116, 117]. In short, the interaction between ROS and cfDNA forms a positive feedback loop, thus amplifying the inflammatory response and aggravating tissue damage.

As the main components of NETs, cfDNA and histones can activate the immune response and help the host to fight against infectious and non‐infectious inflammation. However, excessive release of NETs not only causes cell death, but also triggers physiological processes such as coagulation, inflammatory response, cell death, and impairment of fibrinolysis [118, 119, 120, 121, 122]. Activation of AIM2 inflammatory vesicles by cfDNA from NETs is an immune mechanism leading to the matrix metalloproteinase (MMP) activation of plaque destabilization, which ultimately leads to atherosclerotic thrombosis and arterial embolism [123, 124, 125, 126, 127, 128, 129]. Furthermore, cfDNA‐antibody complexes directly promote B‐cell proliferation and plasma cell differentiation, unveiling its ability in driving both innate and adaptive immunity [96, 119, 130, 131, 132, 133, 134, 135, 136, 137]. Besides, the double‐stranded conformation and high negative charge of cfDNA enable interactions with coagulation factors XII and XI, contributing to coagulopathy disorders [138, 139, 140, 141, 142, 143, 144]. In critical illness, such as sepsis, cfDNA‐fibrin complexes inhibit fibrinolysis by binding both fibrinolytic enzymes and fibrin matrices [145].

Consequently, cfDNA is closely associated with inflammation, involving multiple inflammatory immune signaling pathways such as TLR9, cGAS‐STING, etc. It is also strongly linked to ROS production and NET formation, collectively contributing to inflammatory responses and promoting disease progression.

## 3 cfDNA as a Biomarker for Inflammatory Disorders

### 3.1 Clinical Studies of cfDNA as a Biomarker for Inflammatory Diseases

Immune abnormality and overwhelming inflammation are the major contributors to numerous inflammatory diseases [146, 147, 148, 149, 150, 151]. With the in‐depth elucidation on the mechanism of disease, cfDNA has been proven to be not only a biomarker for inflammatory disorders, but also deeply involved in the development of immune responses, proposing a novel prospective for the diagnosis and treatment of diseases [152, 153, 154, 155]. For example, a clinical study by Vrablicova et al. [156] showed that IBD patients had higher serum cfDNA (both ncDNA and mtDNA) levels compared to healthy individuals. Furthermore, cfDNA can activate inflammatory responses, such as those involving NOD‐like receptor family pyrin domain containing 3 (NLRP3) inflammasome and neutrophils, via the TLR9 receptor. Another study reported that the disease severity in IBD patients was positively correlated with serum levels of cfDNA, which was also strongly correlated with the expressions of colonic TLR9, tumor necrosis factor α (TNF‐α), iNOS, and F4/80 [157].

RA patients also produced more cfDNA in their synovial fluid compared to healthy volunteers [158, 159]. Consistently, cfDNA exacerbated collagen‐induced arthritis (CIA), with significant up‐regulating pro‐inflammatory cytokines (including IL‐6, TNF‐α, and IL‐1β) and disease activity markers (such as C‐reactive protein, CRP) in the serum of RA patients. Luo et al. [159] highlighted that the cfDNA accumulation in the serum of RA patients increased with age and was regulated by the nucleic acid exonuclease three prime repair exonuclease 1 (TREX1).

Clinical studies by Dawulieti et al. [160]showed that sepsis patients tended to have elevated levels of circulating cfDNA released from pathogens and infected host cells. This triggered a macrophage‐mediated pro‐inflammatory response through the TLR9‐MyD88‐NF‐κB signaling pathway. Another report indicated that cfDNA, including nDNA and mtDNA, was elevated early (within one week) in patients with post‐surgical sepsis. This was primarily due to intracellular PRRs recognizing cfDNA as an endogenous DAMP, thereby triggering inflammation through the TLR9 receptor [161, 162]. Furthermore, mtDNA was an important component that induced inflammation in sepsis‐associated lung injury.

In the skin system, Beranek et al. [163] indicated that the cfDNA level was higher in the serum of psoriasis patients compared to healthy controls. The inflammatory and hyperproliferative features of psoriasis were associated with elevated cfDNA levels, which were also positively correlated with levels of different inflammatory biomarkers such as CRP, interferon‐γ (IFN‐γ), TNF‐α, and IL‐6 [164]. cfDNA is also closely related to oral diseases. Huang et al. [165] found that concentrations of salivary and serum cfDNA were positively correlated with the severity of periodontitis, with salivary cfDNA exhibiting a stronger correlation. Furthermore, TLR9 regulates the immune response against periodontal pathogens. Here, endogenous nDNA and mtDNA released by damaged host cells, along with exogenous bacterial or viral DNA, function as ligands for TLR9 [164]. In a cross‐sectional study, plasma cfDNA level in periodontitis patients was significantly higher than that in the healthy group. Other inflammatory mediators in periodontitis, such as IL‐1β, IL‐6, and TNF‐α, are secreted into the bloodstream to activate the innate immune response, thereby releasing DAMP‐derived cfDNA to aggravate tissue destruction in other organs [166]. Additionally, studies strongly suggested that the release of cfDNA is significantly associated with a wide range of inflammatory diseases or responses in various organs throughout the body. In a study of atherosclerosis, Cao et al. [167] identified that inflammatory vesicles mediated by cfDNA were initiators of the inflammatory cascade, causing atherosclerotic plaque degradation and thrombosis. A study on human lip muscle samples after surgical trauma evaluated the relationship among muscle fibrosis, regeneration, and macrophages, and demonstrated that the elevated cfDNA level after injury correlated with the pro‐inflammatory macrophages. Trujillo et al. [52] found that in two independent cohorts of idiopathic pulmonary fibrosis (IPF) patients, plasma mtDNA was abnormally increased and could specifically activate TLR9, which was associated with the deterioration of transplant‐free survival. Chen et al. [95] revealed that NETs‐DNA promoted NF‐κB‐dependent autoimmune responses in chronic obstructive pulmonary disease (COPD) by activating the cGAS or TLR9 pathway. In nasal secretions of eosinophilic chronic rhinosinusitis (ECRS) patients, the cfDNA level was higher than that in nasal secretions of healthy volunteers, and was positively correlated with the abundance of eosinophil extracellular trap (EET) [168]. Moreover, cfDNA was strongly correlated with cellular damage and inflammatory parameters in liver injury, which was proven to be comparable to alanine aminotransferase (ALT) in predicting mortality [169]. Nishimoto et al. [170] reported that, in obese individuals, the overgrowth of adipocytes caused the release of cfDNA, which in turn induced inflammation in the body, and increased the expression of TLR9 recognition protein, as well as insulin resistance of the organism. In systemic lupus erythematosus (SLE) [49], obesity and stroke [171], the levels of cfDNA were all positively correlated with disease activities. Thus, clinical studies have consistently shown that patients with inflammatory conditions have elevated levels of cfDNA in their biological fluids (such as blood, cerebrospinal fluid, urine, sputum, etc.) compared to healthy controls [172, 173, 174, 175, 176, 177, 178]. Part of the clinical studies corresponding to cfDNA are summarized in Table 1.

**TABLE 1:** Correlation between cfDNA and disease development in clinical samples.

Diseases	Patient numbers	Patient	Analytical results	Involvement in the disease process	Application	Refs.
IBD	Health volunteers (n = 20) IBD patients (n = 52)	Serum	The serum cfDNA level is strongly correlated with the expression levels of colonic TLR9, TNF‐α, iNOS and F4/80.	The expression of TLR9^+^, TNF‐α, iNOS and macrophage markers F4/80 is elevated in IBD disease.	cfDNA‐TLR9 signaling as a target for IBD treatment.	[157]
	Health volunteers (n = 29) IBD patients (n = 100)	Serum	The levels of ncDNA and mtDNA in IBD patients are higher than that in healthy volunteers.	None	Predict clinical remission in IBD patients.	[156]
Liver injury	Health volunteers (n = 40) DILI patients (n = 40) ALD patients (n = 40)	Serum	The cfDNA level in DILI and ALD patients is higher than that in healthy wolunteers, and as a promising biomarker, cfDNA can be used to predict mortality.	cfDNA amplifies liver injury and inflammatory responses through the TLR9 pathway and a neutrophil‐mediated pathway.	Predict the outcome and prognosis in AIL patients.	[169]
Periodontitis	Health volunteers (n = 10) Gingivitis patients (n = 10) Periodontitis patients (n = 10)	Saliva and serum	The levels of cfDNA in saliva and serum of gingivitis patients are significantly higher than those in healthy volunteers, and the correlation in saliva is much stronger than that in serum.	The activity of nanoparticles to clear cfDNA and regulate M1/M2 macrophage phenotypes.	cfDNA‐TLR9 signaling as a target for periodontitis treatment.	[165]
	Healthy volunteers (n = 25) Gingivitis patients (n = 31) periodontitis patients (n = 25)	Gingival sulcus, saliva and plasma	The cfDNA level in patients with periodontal is significantly elevated, and GCF and saliva cfDNA are positively correlated with periodontal parameters.	Unmethylated CpG DNA recognizes TLR9 receptors, generates pro‐inflammatory responses through MyD88, and activates NF‐κB, AP‐1, and mitogen activated protein kinase signaling pathways in activated B cells.	Predict disease severity in periodontitis patients.	[166]
Atherosclerosis	Healthy volunteers (n = 18) ECRS patients (n = 24)	Serum	The serum cfDNA concentration increase transiently during the acute phase after stroke and myocardial infarction, confirming that inflammatory vesicle activation is mediated by cfDNA.	cfDNA activates AIM2 inflammasomes via the TLR9 signaling pathway, leading to plaque instability through the activation of matrix metalloproteinases.	Predict postoperative remission in ECRS patients.	[167]
Sinusitis	Healthy volunteers (n = 20) CRSwNP patients (n = 20)	Nasal secretion	Compared to healthy volunteers, ECRS patients have elevated cfDNA concentration in nasal secretion and is positive correlated with the abundance of EETs.	cfDNA is involved in numerous inflammations related signaling pathways, such as TLR, NF‐κB, IRFs, and MAPKs, leading to the production of a range of pro‐inflammatory cytokines and chemokines.	cfDNA‐TLR9 signaling as a target for ECRS treatment.	[168]
Sinusitis with nasal polyps	Healthy volunteers (n = 20) CRSwNP patients (n = 20)	Nasal secretion	The level of cfDNA in nasal secretion increases and is significantly positively correlated with the degree of airway inflammation.	Reduce cfDNA levels, inhibit TLR 9 activation and suppress NET formation.	cfDNA‐TLR9 signaling as a target for CRSwNP treatment.	[771]
RA	Healthy volunteers (n = 51) RA patients (n = 108)	Serum	The level of cfDNA in serum is significantly increased in RA patients.	Through the TLR9 signaling pathway, patients exhibited an upward trend in M1‐type macrophages, IL‐6, TNF, IL‐1β, MMPs, and DAI levels.	cfDNA‐TLR9 signaling as a target for RA treatment.	[158]
	Healthy children (n = 12) Healthy elderly (n = 45) Elderly RA patients (n = 26)	Serum	Compared with healthy children, the serum cfDNA concentration is significantly elevated in healthy elderly individuals and elderly RA patients. Moreover, elderly RA patients have even higher cfDNA accumulation than healthy elderly individuals.	The level of cfDNA fragments increases with age, and the accumulation of cfDNA fragments may activate the cGAS signaling cascade. Addition, high levels of TREX1 expression can reduce excess DNA fragments.	cfDNA‐cGAS as a target for RA treatment.	[159]
Systemic lupus erythematosus	Healthy controls (n = 43), SLE patients (n = 54)	Plasma	Plasma cfDNA concentration in the SLE group is significantly higher than that in healthy controls.	cfDNA is significantly correlated with albumin, Ccr, and quantitative 24‐hour urinary protein levels, and positively correlated with LDG and neutrophil levels.	cfDNA as a target for SLE treatment.	[49]
Ischemic stroke	Patients before and after 90 days of treatment (n = 90)	Plasma	The plasma cfDNA concentration increases in patients with poor clinical outcomes.	None	Predict the outcome in vessel occlusion after stroke patients.	[171]
Sepsis	Healthy volunteers (n = 16) Septic patients (n = 15)	Serum	The serum cfDNA level in sepsis patients is significantly higher than that in healthy volunteers.	cfDNA induces TLR9 activation and TNF‐α secretion.	cfDNA‐TLR9 signaling as a target for sepsis treatment.	[160]
	Patients with rapid recovery (n = 6) Patients with CCI (n = 41)	Plasma	The levels of nuDNA and mtDNA in sepsis patients are higher than that in healthy controls.	None	Predict the outcome in sepsis patients.	[161]
	Healthy volunteers (n = 16) Severe sepsis patients (n = 30)	Plasma	The plasma cfDNA concentration is significantly elevated in sepsis patients.	cfDNA recognizes TLR receptors, triggers an intracellular signal transduction cascade, activates NF‐κB pathway and triggers the expression of inflammatory cytokines.	cfDNA‐TLR9 signaling as a target for sepsis treatment.	[162]
Psoriasis	Healthy volunteers (n = 20) Psoriasis patients (n = 30)	Serum	The serum cfDNA level in psoriasis patients is significantly higher than that in patients with the common forms prior to the treatment.	The inflammatory biomarkers of CRP, TNF‐α, IL‐6 and leptin are elevated in psoriasis patients.	cfDNA as a target for psoriasis treatment.	[164]
	Healthy volunteers (n = 22) Psoriasis patients (n = 28)	Serum	The serum cfDNA level in psoriasis patients is significantly higher than that in healthy volunteers.	None	cfDNA as a target for psoriasis treatment.	[163]

Abbreviations: AIL, angiocentric immunoproliferative lesions; AIM2, activation of melanoma 2 inflammatory vesicles; ALD, alcoholic liver disease; ALT, alanine aminotransferase; AP‐1, activator protein 1; CCI, charlson comorbidity index; Ccr, creatinine clearance rate; CCR,; cGAS, cyclic GMP‐AMP synthase; CpG, cytidylyl phosphate guanosine; CRP, c‐reactive protein; CRSwNP, chronic rhinosinusitis with nasal polyps; DAI, disease activity index; DILI, Drug‐induced liver injury; ECRS, eosinophilic chronic rhinosinusitis; EET, eosinophil extracellular trap; F4/80, mouse EGF‐like module‐containing mucin‐like hormone receptor‐like 1; GCF, gingival crevicular fluid; IBD, inflammation bowel disease; IL‐1β:interleukin – 1β;IL‐6, interleukin‐6; IL‐8, interleukin‐8; IRFs, interferon regulatory factors; IFN‐γ, interferon gamma; iNOS, inducible nitric oxide synthase; LDG, low‐density granulocyte; LN:lupus nephritis; MAPKs, mitogen‐activated protein kinases; MMPs, matrix metalloproteinases; MyD88, Myeloid differentiation primary response protein 88; NET, neutrophil extracellular trap; NF‐κB, nuclear factor κ light chain enhancer; RA, rheumatoid arthritis; SLE, systemic lupus erythematosus; TLRs, toll‐like receptors; TNF‐α, tumour necrosis factor‐α; TREX1, three prime repair exonuclease 1.

### 3.2 Clinical Detection Technology for cfDNA

**FIGURE 5:**
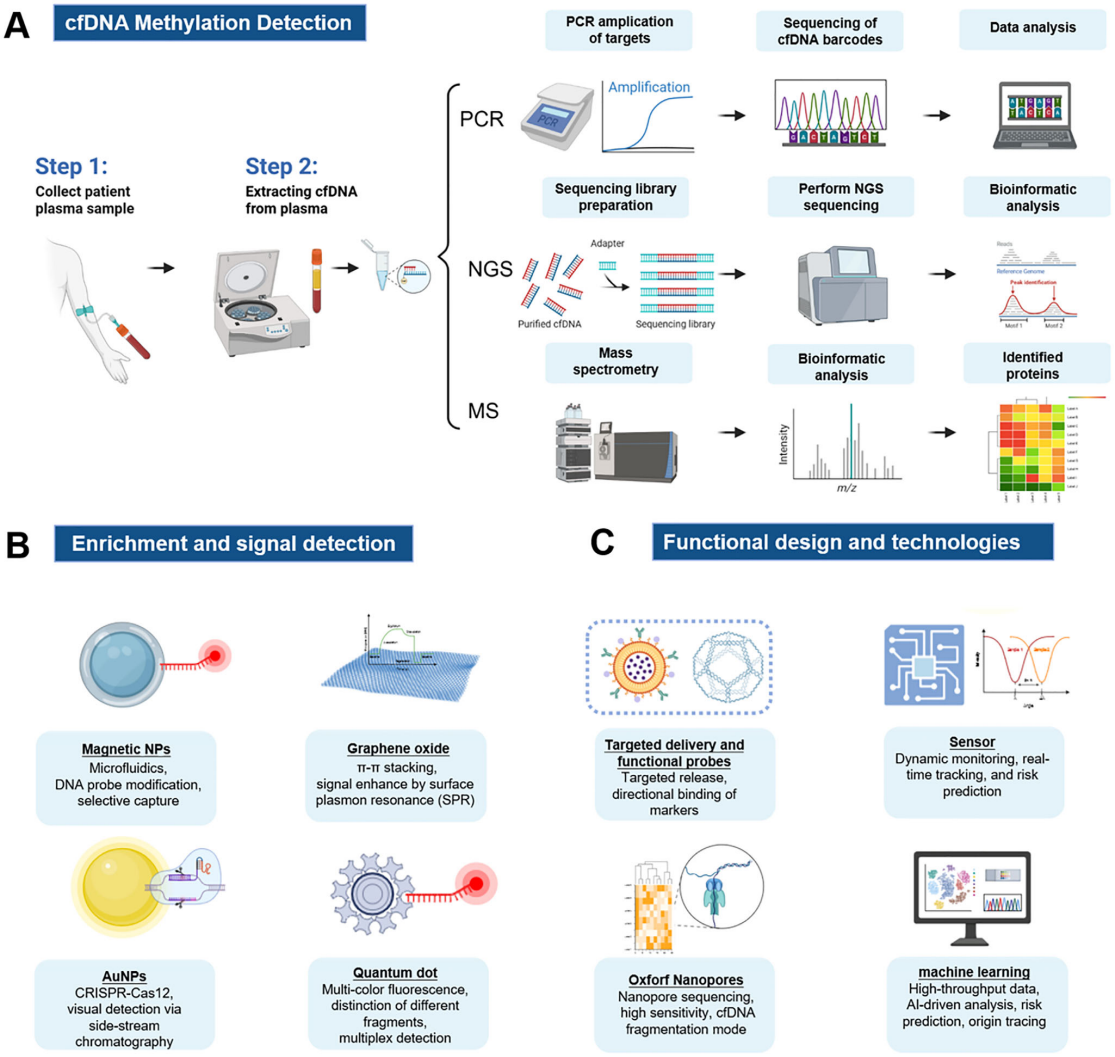
Detection technologies for cfDNA. (A) Current approaches for cfDNA detection. Figure was created with BioRender.com. (B) Typical nanotechnology‐driven detection on cfDNA using DNA‐functionalized Au nanoparticles as probes [179]. Copyright 2021 American Chemical Society. (C) Schematics of CRISPR‐based wearable system for the extraction and real‐time monitoring of cfDNA in vivo in a minimally invasive manner [180]. Copyright 2022, Springer Nature.

Current clinical approaches for cfDNA detection mainly encompass polymerase chain reaction (PCR), next‐generation sequencing (NGS), analysis of methylation patterns, and mass spectrometry (MS) [181, 182, 183, 184, 185, 186, 187] (Figure 5). PCR method is widely used in molecular biology, owing to its high sensitivity and specificity. Its basic principle is to selectively amplify the target DNA sequence with specific primers [188, 189, 190, 191, 192, 193, 194]. Via repeated cycles of denaturation, annealing, and extension, PCR can increase target DNA copies to millions in a short time. PCR amplification on the specific sequences of cfDNA helps monitor disease presence, progression, and therapeutic response, providing the basis for individualized treatment [195, 196, 197, 198]. However, the heterogeneous molecular lengths, low plasma concentration, and high sequence similarity with healthy human DNA poses significant challenges for the detection and analysis of cfDNA [199, 200, 201, 202, 203, 204, 205]. To achieve higher clinical sensitivity, it is necessary to simultaneously incorporate multiple molecular markers, which greatly undermines the strengths of PCR.

NGS is a high‐throughput sequencing technology capable of generating large amounts of DNA sequence data in a short period of time [206, 207, 208, 209, 210, 211, 212, 213]. NGS technology can comprehensively analyze cfDNA and provide rich genetic information [214, 215, 216, 217, 218, 219, 220, 221, 222]. It is also used to screen for genetic diseases by identifying mutations associated with genetic diseases through whole genome sequencing of cfDNA, demonstrating great potential in the fields of tumor surveillance, non‐invasive prenatal screening, and genetic disease detection [223, 224, 225, 226, 227, 228, 229, 230, 231, 232, 233, 234, 235, 236, 237]. Although NGS‐based approaches hold promise for the clinical detection, the need for quality controls and validation strategies in clinical metagenomics must be addressed.

Methylation analysis is a promising method to study DNA methylation patterns, helping us to understand the mechanisms of gene regulation associated with disease [238, 239, 240, 241, 242, 243, 244, 245, 246, 247, 248, 249, 250, 251, 252, 253]. Emerging single‐molecule sequencing technologies will further improve the sensitivity and accuracy of methylation detection [254, 255, 256, 257, 258, 259, 260, 261, 262, 263, 264, 265]. More excitingly, the combination of multiple histological data (e.g. transcriptome and proteome) will deepen our understanding on cfDNA methylation patterns.

MS, as a highly sensitive and high‐resolution analytical technique, is a useful tool for cfDNA detection at very low concentrations [266, 267, 268, 269, 270, 271, 272, 273, 274, 275, 276, 277, 278, 279]. By analyzing the mass spectrogram, structural information, molecular weight and possible modifications, high‐resolution data are obtained, thus providing more comprehensive and precise information for early detection and monitoring of the disease.

### 3.3 Advances in cfDNA‐Based Diagnostics of cfDNA

Technological advancements in MS and NGS have significantly enhanced the analysis of proteogenomic signatures in blood. However, only a few blood‐based cancer biomarker assays have been approved by the FDA [280, 281, 282, 283, 284, 285]. Chip‐based platforms that utilizing electronic or electrochemical detection are emerging as promising solutions for clinical sample analysis, owing to their compatibility with automation and the development of cost‐effective diagnostic devices [286, 287, 288, 289, 290, 291]. Electrochemical cfDNA biosensors can convert the target cfDNA reaction into measurable electronic signals. This process typically relies on capture probes immobilized on the surface of electrodes that capture cfDNA as a specific sequence [292, 293, 294, 295, 296, 297, 298, 299]. Meanwhile, the signal probe employs an electrochemical tag to generate a detectable signal, which facilitates sensitive detection of cfDNA [300, 301, 302, 303, 304, 305]. Electrochemical‐based DNA biosensors allow for rapid and highly sensitive cfDNA analysis, providing new tools for early cancer detection, genetic disease screening, and personalized medicine [306, 307]. Li et al. [308] designed a high‐performance electrochemical biosensor using the rolling circle amplification (RCA) technique, with doxorubicin (DOX)‐loaded gold nanoparticles (Au NPs or Au) as a tag. This sensor demonstrated enhanced signal intensity and efficient signal amplification, significantly improving detection sensitivity, which was particularly crucial for low‐abundance cfDNA analysis.

Recently, nanotechnology‐driven cfDNA detection exhibit huge exceptional accuracy, molecular recognition capabilities, and accelerated signal transduction kinetics [179, 309]. Notably, nanomaterials such as Au NPs, quantum dots and iron oxide nanocrystals, etc, have played critical roles in the development of biosensors due to their unique optical, structural, magnetic and electronic properties [310]. These NPs possess a large specific surface area and are capable of interacting with target molecules more efficiently, thereby enhancing detection sensitivity [311]. For example, Au NP can be used in analytical methods such as surface‐enhanced Raman scattering (SERS) owing to their excellent optical properties, while quantum dots can be used in fluorescence detection because of their distinctive fluorescence properties [312]. Choi et al. [179] developed a CRISPR‐Cas12a—based nucleic acid amplification‐free fluorescent biosensor with DNA—functionalized Au NP for efficient cfDNA detection. Its working principle relied on the metal‐enhanced fluorescence (MEF) effect. Specifically, target cfDNA activated the CRISPR‐Cas12a complex, degrading the bounded single‐stranded DNA (ssDNA). The change in the distance between the functionalized Au NP and the fluorophore altered the fluorescence emission properties, changing the fluorescence color from purple to reddish purple and enabling qualitative and quantitative cfDNA analysis [313]. Additionally, Senapati et al. [314] proposed an economical and highly sensitive technique for cfDNA detection. This technique employs a tumor‐specific mutation bridging magnetic NP coupling strategy to isolate and enrich target DNA complexes. It also enhances binding efficiency to cfDNA by modulating the surface properties of these NPs.

As a wearable detection device, microneedles can extract targets from tissue fluids and analyze them in a minimally invasive manner. However, some microneedles are limited in their ability to provide real‐time monitoring for macromolecule biomarkers [315, 316, 317, 318, 319, 320]. Kong et al. [180] reported a CRISPR‐activated graphene biointerface and demonstrated that this synergistic effect was integrated into wearable microneedles for in vivo extraction and long‐term monitoring of extracellular cfDNA, holding great promise for early disease screening and prognosis.

Despite significant advancements in high‐throughput sequencing instruments, library preparation methods, and bio‐informatics pipelines, cfDNA diagnostics have not yet been widely adopted in clinical practice. Significant foundational research efforts are critically required to standardize cfDNA detection, and develop unified analytical and reporting frameworks, including type‐specific isolation methodologies, standardized reference materials, assay parameters, and optimization of specificity and sensitivity thresholds. Addressing these challenges will enhance the clinical utility in prognostic assessment and therapeutic efficacy evaluation.

## 4 Drug Discovery Targeting cfDNA‐Based Interventions

### 4.1 Drug Discovery Strategies for cfDNA‐Based Interventions

**FIGURE 6:**
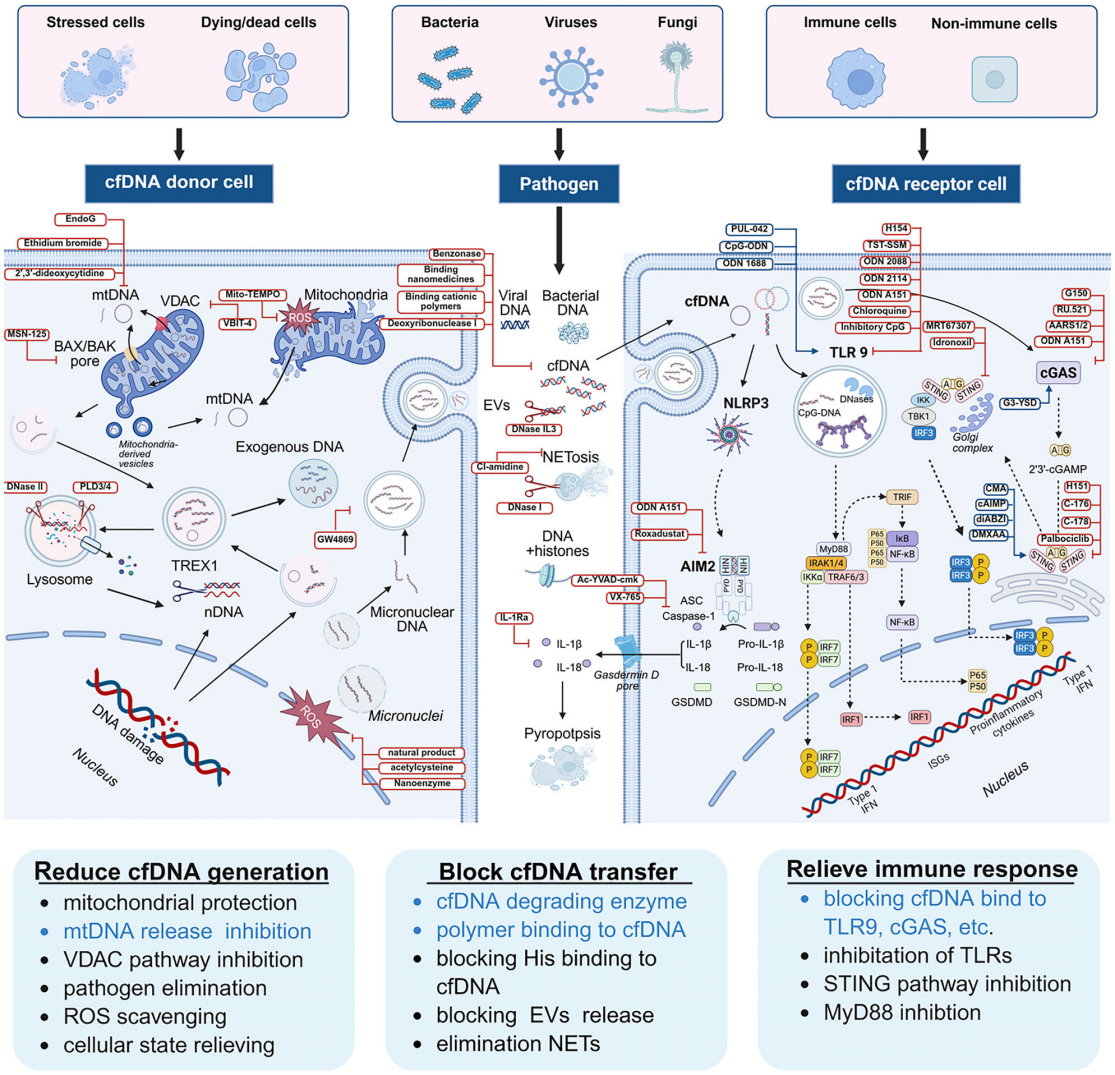
Intervention strategies targeting the generation, transport, and immune activation pathways of cfDNA. Inhibition of cfDNA release and removal of nucleic acids from the source are the effective strategies for blocking nucleic acid sensor activation. Cl‐amidine can be applied to inhibit the formation of NETs. MSN‐125 can inhibit mitochondrial DNA leakage, in addition to the inhibitory effects of cyclosporin A and VBIT4. To remove nucleic acid, nuclease (e.g., EndoG, DNase I, and DNase II) and nanomedicines can be applied to reduce extracellular DNA, whereas ethidium bromide and 2′,3′‐dideoxycytidine can be used to remove intracellular mitochondrial DNA. Targeted intervention in DNA‐sensing pathways can help prevent inflammatory disease. Critical proteins in different DNA‐sensing pathways, such as TLR9, cGAS, STING, TBK1/IKK, AIM2, caspase‐1, and IL‐1I, can be effectively regulated by pharmacological inhibitors (red border) or activators (blue border). Abbreviations: Ac‐YVAD‐cmk, N‐acetyl‐tyrosyl‐valyl‐alanyl‐aspartyl chloromethyl ketone; cAIMP, cyclic adenosine‐inosine monophosphate; CMA, 10‐carboxymethyl‐9‐acridanone; G3‐YSD, G3‐ended Y‐form short DNA; IL‐1Ra, interleukin‐1 receptor antagonist; TST‐SSM, thiostrepton encapsulated in phospholipid sterically stabilized micelles. Figure was created with http://www.BioRender.com.

Based on the physical, biochemical, and immune properties of cfDNA, inhibiting the generation of cfDNA, scavenging the existing cfDNA, and inhibiting its activation of the downstream inflammatory pathways are expected to modulate the immune response and inhibit the pro – inflammatory cascade [321, 322, 323, 324, 325, 326, 327] (Figure 6). Here, we review the present knowledge related to cfDNA interventions for inflammation manipulation. Table 2 provides a comprehensive list of agents targeting cfDNA‐driven inflammation, categorized by their clearance strategy, drug targets, and specific blocking mechanisms. First, drugs can be developed by targeting the donor cells and blocking the generation of cfDNA, which is mainly derived from cell death, including apoptosis, necrosis, and NETs formations, as well as other pathways such as active secretion [328, 329, 330, 331, 332, 333]. Considering the transport of cfDNA, it can be degraded once released from the original organelles and/or host cells, where nucleic acid endonucleases, specifically deoxyribonuclease (DNases) can digest single‐ or double‐stranded DNA and are responsible for delegating cfDNA [334, 335, 336, 337, 338, 339, 340, 341]. Furthermore, DNA enriched in phosphate groups, is negatively charged in physiological conditions. Through electrostatic interaction, positively charged cationic substances will bind to DNA to form complexes, thus contributing to the clearance of cfDNA [342, 343, 344, 345, 346, 347, 348, 349, 350]. Positively charged cationic polymers, including polyamidoamine dendrimer (PAMAM) [351], polyethyleneimine (PEI) [352], polyvinylpyrrolidone (PVP) [353], poly‐L‐lysine (PLL) [354], polycation hexadimethrine bromide (HDMBr) [355], and poly (ethylene glycol) methacrylate (PDMA) [356], which are commonly served as nonviral nucleic acid carriers, can effectively neutralize the negative charge of DNA, reducing the repulsive forces between DNA molecules and forming cohesive complexes. As a consequence, the design of therapeutic agents targeting the removal of cfDNA can directly apply cationic polymers, which are conducive to the clearance and degradation of cfDNA, allowing the regulation of the intracellular DNA concentration [357, 358]. More importantly, cationic polymers show the characteristics of low cost, simple preparation, high cellular internalization, and favorable binding efficiency on nucleic acid. Nic polymers exhibit several advantageous properties, including low cost, straightforward preparation, efficient cellular internalization, and strong binding affinity for nucleic acids. Nevertheless, their biomedical applications are restricted by the potential cytotoxicity. Surface modification offers a novel strategy for binding cfDNA via electrostatic attraction [359, 360, 361, 362, 363, 364, 365]. It is worth mentioning that charge‐modified nanomaterials have been frequently designed to be capable of exposing cations under specific conditions [366, 367, 368, 369]. In inflammation, the cationic portion of the nanomedicine is exposed to bind cfDNA, improving their targeting and specificity, while reducing the side‐effects at non‐targeted sites [370, 371, 372, 373, 374].

**TABLE 2:** Drug discovery strategies targeting cfDNA removal.

cfDNA clearance strategy		Drug targets	Specific blocking process	Refs.
Inhibiting cfDNA generation	Reducing cell apoptosis	Ly6G‐specific antibodies deplet neutrophils and block PAD4 molecules to inhibition NETosis.	Inhibition of NETosis, neutralisation of cfDNA and inhibition of inflammatory vesicle activation effectively can effectively prevent recurrent vascular events after stroke.	[167]
		High‐density helical peptide molecular clips based on dendritic cationic polymer	Guanidinium‐functionalized peptides achieve efficient capture and stable clearance of cfDNA through electrostatic attraction, salt bridging, and spatial confinement of rigid alpha helices, and effectively inhibit abnormal activation of TLR9.	[377]
		DIDS, acting as an VDAC 1 oligomerisation inhibitor	Reduce mitochondrial oxidative stress and DNA damage, protect mitochondrial complex proteins to combat DDP‐induced apoptosis.	[779]
		Neuroprotective efficacy of tetrahedral backbone nucleic acids against neuronal apoptosis in ischaemic stroke, reversing neuronal loss and attenuating apoptosis.	Blocking the TLR 2‐MyD 88‐NF‐κB signalling pathway.	[780]
		The small molecule inhibitor of Bax oligomerisation, MSN‐125, allows cells to escape apoptotic stimuli and rescues neurons from death after excitotoxicity.	Mitochondrial outer membrane permeabilisation triggered by oligomerisation of the Bcl‐2 family protein Bax/Bak is an irreversible step leading to apoptosis.	[781]
	Preventing cell necrosis	The small molecule BAI inhibitor BAI1 inhibits conformational changes during BAX activation, preventing mitochondrial translocation and oligomerisation of BAX.	Blocking BAK/BAX pathways, inhibiting the release of mtDNA caused by miMOMP in senescent cells, thereby inhibiting the cGAS‐STING pathway and finally delaying cellular aging.	[401]
		TA modified negatively charged MHT can effectively targeting CIRI tissues and neuronal mitochondria.	Reduce the release of mtDNA and the production of mtROS.	[782]
		Inhibition ZBP‐1 derived PANotosis or knockout of ZBP 1.	Preventing cell necrosis by disrupting the formation of dsRNA in DNA damage.	[783]
		Binding of the pro‐inflammatory cytokine TNF to pan‐cysteinyl asparaginase inhibitors activates RIPK3‐dependent necrotic apoptosis in certain cell types.	TLR agonist, activates TLR4 and TLR3 signalling and also stimulates RIPK3‐dependent necrotic apoptosis in the presence of pan‐cysteinyl asparaginase inhibitors.	[784]
		Integration of silver nanoclusters and tumour necrosis factor‐alpha antibodies into DNA hydrogels to inhibit bacterial growth.	DNA hydrogel slow‐release TNF‐α antibody that effectively blocks TNF‐α and promotes macrophage M2 polarisation.	[785]
	Inhibiting cell active secretion	The positively charged residues in the N‐terminal structural domain of VDAC1 interact with mtDNA, promoting the oligomerization of VDAC1.	VDAC oligomerization inhibitor VBIT‐4 reduces mtDNA release, IFN signaling, and NET formation in a murine model of systemic lupuserythematosus.	[472]
		Alpha‐linolenic acid attenuates lung injury by inhibiting pyridine inflammatory vesicle‐driven activation of NET‐induced macrophage sepsis.	Inhibits the formation of NETs at the source and inhibits the active secretion of relevant cytokines by the cells.	[786]
		TGF‐β induces the release of mitochondrial DNA from healthy HSC through voltage‐dependent anion channels.	Stimulated activation of the cGAS‐STING‐IRF 3 pathway	[787]
		GW 4869, an inhibitor of intracellular vesicle formation, significantly reduced the production of mt‐cfDNA.	The proteasomal mitochondrial quality control system is upstream of mt‐cfDNA secretion and autophagy plays a role in the cellular digestion of mitochondrial DNA in the cytoplasm.	[788]
		Targeting NET with DNase I or CI‐amidine ameliorated psoriasis and reduced expression of IL‐17 A, lipid transport protein 2 and IL‐36 G.	Activation of neutrophils leads to the release of NETs, NETs drive inflammatory responses in the skin by activating epidermal TLR 4/IL‐36 R crosstalk.	[789]
		TFAM acts as a selective autophagy receptor, binds to LC3 and transports mtDNA to autophagic lysosomes for degradation.	Mitochondrial autophagy can be induced by damaging mitochondrial stress.	[790]
Direct cfDNA clearance	DNase I	Combination of DNase I and NAC protect liver damage in AILI	Reduce the elevation of circulating TNF – α, IL‐6, MCP‐1, and HMGB1, decrease liver neutrophils, and partially restore the reduction of Kupffer cells.	[169]
		DNase I significantly reduces expression of apoptosis‐critical proteins and terminal deoxynucleotidyl transferase‐mediated nick end labelling in apoptotic hepatocytes.	Inhibit neutrophil infiltration and suppress the NLRP3 signaling pathway.	[458]
		TGF‐β signalling inhibitor LY 2157299 reduces AIC‐induced liver metastases and peritoneal metastases in nude mice with NETs.	Targeted inhibition of downstream effectors of NET, such as TGF‐β signaling.	[459]
		The hybrid protein NM was prepared by glutaraldehyde‐mediated cross‐linking of human serum albumin and DNase‐I at optimised molar ratios.	Targeted lung cf‐mtDNA clearance.	[460]
		Cross‐linking of carboxymethyl chitosan with DNAzyme‐functionalized OHA further enhances the adhesion of DHA.	Hydrolyze plasmid DNA and disrupt the structure of NETs.	[461]
		EndoG is a mitochondrial endonuclease.	EndoG is responsible for degrading damaged mtDNA.	[791]
	Artificial DNase I	Polyimidazole efficiently attacks the phosphodiester bonds of NAs and cleaves them into small fragments.	Polyethylene glycolated polyimidazole as a mimetic DNA enzyme effectively mitigates its own DNA.	[473]
Targeted clearance of cfDNA	Nanomedicines	MONs can bind and scavenge ROS, and PEI25k couples to the surface of MONs, giving it good stability and showing high binding affinity for ct‐DNA.	Inhibition of the TLR9 pathway attenuates cfDNA‐ and ROS‐mediated inflammatory responses.	[157]
		Anti‐inflammatory macrophage‐derived exosomes as chassis with positively charged oligo‐lysine modified on the membrane and electrostatically bound to cfDNA.	Removal of cfDNA, inhibition of the TLR9 pathway and polarisation of macrophages.	[158]
		PEG‐TK‐NPArg has a strong competitive affinity and binds to ctDNA due to positive charge exposure upon ROS‐triggered deshielding of the PEG outer layer.	Binds to ctDNA and effectively inhibits TLR9 activation.	[536]
		TA can interact with DNA phosphate bonds via hydrogen bonds to form a stable tannic acid‐DNA complex that scavenges ROS and cfDNA.	TMP‐NP had good antimicrobial activity and also cleared multiple inflammatory mediators, including LPS, cfDNA and ROS.	[541]
		Self‐assembled formation of cationic nanoparticles exhibits better DNA binding affinity in vitro.	cNPs can be internalised into endosomes and disrupt the interaction between cfDNA and TLR9.	[530]
		PEI electrostatically attracts cfDNA and tetrasulfide bonds consume ROS via redox reactions.	Elimination of LPS, ROS and cfDNA from the inflammatory microenvironment and reduction of oxidative stress.	[733]
	Cationic polymers	PAMAM‐G3 positively charged coating on the surface of selenium‐containing hydroxyapatite nanoparticles SeHAN.	Binding to scavenge cfDNA, thereby inhibiting the activation of TLR9.	[165]
		Scavengers with longer backbones and higher charge density preferentially accumulate in inflamed joints of arthritic rats.	Electrostatic binding of cfDNA and inhibition of TLR signalling pathway.	[529]
		Amino and hydroxyl groups on the surface of chitosan form hydrogen bonds with cfDNA, which leads to binding and removal.	Cationic polymers with strong DNA binding capacity to remove cfDNA from the dermis.	[532]
		Regulation of the number of grafted generations of degradable PAMAM molecules followed by electrostatic removal of cfDNA.	Inhibition of TLR9 activation by cfDNA in immune cells.	[739]
		HDBr is a positively charged nanoscale compound that condenses DNA into nanocomplexes for delivery into cells.	Clears mtDNA and blocks the TLR9 signalling pathway.	[557]
Targeting cfDNA recognition receptors	Blocking cthe TLR pathway	Functional two‐dimensional nanoplatforms with flexible linear structure and higher cfDNA affinity than dendrimer‐coated polyglyceramide nanosheets.	TLPGA effectively adsorbs and removes cfDNA from inflammatory sites, thus further inhibiting cfDNA‐induced TLR9 activation and EETs formation.	[168]
		Cationic polyglycerol‐modified BP nanosheets with superior cfDNA scavenging ability.	Inhibited the activation of TLR9, which in turn reduced the formation of NETs, reduced cfDNA.	[771]
		Cationic NPs reduced serum cfDNA, ROS, activated macrophages, pro‐inflammatory cytokine levels.	Co‐localisation of CpG DNA and TLR9 in the cytosol induces recruitment of the articulin MYD88 to initiate signalling.	[162]
		These negatively charged molecules are captured by electrostatic interaction using cationic O‐P hydrogels.	Reduction of cfDNA levels, downregulation of TLR4/9‐NF‐κB pathway activation.	[757]
		Microsphere assembly of PDA@GM MSs is based on adjacent nucleotide pairs on the DNA biallelic axis and can capture stored cfDNA.	Expression of cfDNA‐associated TLR 9 was significantly reduced, and ROS levels were also significantly reduced.	[792]
		HMGB1 interacts with mtDNA as required for robust activation of the innate immune response to nucleic acids in autoimmune diseases.	Inhibition binding of HMGB 1 to mtDNA during hypoxia inhibits activation of the TLR 9 signalling pathway.	[793]
		MtDNA encodes a formylated peptide, which can activate the TLR response to this pathogen‐associated molecular pattern.	Inhibition of release of mitochondrial DNA to avoid neutrophil‐mediated organ damage induced by TLR9 activation.	[453]
		Chloroquine administration decreases the anti‐inflammatory cytokines TNF‐α and IL‐10 and reduces the abundance of TLR9 proteins in the spleen.	Chloroquine reduced the abundance of TLR9 protein in the spleen, and phosphorothioate oligodeoxynucleotide H154 mirrored chloroquine in all functional parameters.	[794]
		Nanomedicines formed by encapsulating TST in phospholipid space‐stable micelles reduce bacterial load and pro‐inflammatory cytokine levels in blood and peritoneal lavage fluid.	Thiostreptolysin is a TLR 7–9 Inhibitor.	[795]
		CpG‐ODN increased TLR 9 activation and p38 phosphorylation in the myocardium, and p38 mitogen‐activated protein kinase inhibition eliminated CpG‐ODN‐induced cardiac injury.	CpG‐ODN, a ligand for TLR 9, promotes I/R‐induced cardiomyocyte apoptosis and caspase‐3 cleavage levels in the myocardium.	[796]
		PUL‐042 is a clinical‐stage inhaled drug that contains synthetic ligands for Toll‐like receptor 2/6 (Pam 2CSK 4) and TLR 9 (ODN M362).	PUL‐042 is a clinical‐stage aerosol drug consisting of synthetic ligands for Toll‐like receptors (TLR) 2/6 and TLR 9.	[797]
		Excitatory hippocampal CA1 neuron clusters show persistent double‐stranded DNA breaks after hours of learning.	Neuron‐specific knockdown of TLR 9 impairs memory while blocking gene expression changes in specific excitatory CA1 neuron clusters.	[798]
	Blocking the cGAS pathway	3’→5’ DNA exonuclease TREX1 reduces the accumulation of DNA fragments.	Removes the accumulation of DNA fragments in vivo and inhibits the activation of cGAS/STING signalling pathway.	[159]
		Platinum‐doped positively charged carbon dots Pt‐CDs bind to extracellular cfDNA and inhibit swollen double‐stranded DNA‐induced chemokine expression.	Removes extracellular cfDNA, inhibits over‐activation of the cGAS‐STING pathway.	[721]
		Loading cationic nanoparticles cRNPs into PEG hydrogels to form injectable nanodrug‐hydrogel complexes.	cRNP removes cGAS‐activated DNA and delivers cGAS inhibitors for effective cGAS inhibition.	[738]
		The methyl pyrazole ring and the hydroxyacetyl group were used as linkage sites to connect the cGAS binding agent.	TH35 efficiently and selectively degrades cGAS and significantly inhibits double‐stranded DNA‐induced cGAS signalling activation.	[489]
		Mitochondrial damage stimulates mtDNA release, inducing production of the second messenger cGAMP and activation of the pro‐inflammatory pathway.	Release of mitochondrial DNA into cytoplasmic lysate and activation of the DNA‐sensitive cGAS/STING pathway in inflammatory injury.	[791]
		The small molecule cGAS inhibitor RU.521 is active and selective in cellular assays of cGAS‐mediated signaling.	Up binding to DNA, cyclic GMP‐AMP synthase (cGAS) produces the cyclic dinucleotide, which leads to the upregulation of inflammatory genes.	[799]
		PAH inhibits cytoplasmic DNA‐induced innate immune response by suppressing cGAS activity.	Polycyclic aromatic hydrocarbons PAHs can effectively suppress cGAS‐STING signaling and can be used to treat cGAS‐mediated autoimmune diseases.	[800]
		Selective inhibition of cGAS/STING with G150/H151 attenuates pulmonary fibrosis after polystyrene microplastics exposure.	Polystyrene microplastics triggers apoptosis and promotes pulmonary fibrosis by activating the cGAS/STING signalling pathway.	[801]
		RU.521 caused a shift in microglia phenotype from M1 to M2, which was eventually eliminated by 2′ 3′ ‐cGAMP.	SAH leads to neuroinflammation caused by microglia activation, and RU.521 modulates microglia polarisation through the cGAS/STING/NF‐κB pathway in early post‐SAH brain injury, improving neurological outcome and reducing neuroinflammation.	[802]
		Using TREX 1‐deficient monocytes, A151 eliminated cGAS activation in response to endogenous accumulation of DNA.	Inhibitory ODN A151 effectively inhibited the activation of cGAS in response to cytoplasmic DNA, thereby inhibiting type I IFN production in human monocytes.	[803]
		MCT1‐mediated high levels of l‐lactate significantly inhibit cGAMP and IFNβ production.	AARS1/2 acts as an L‐lactate sensor and modifies the cGAS lysine site via ATP‐dependent lactoyl transfer.	[382]
	Blocking the STING pathway	NS uses its myocardial targeting properties to specifically bind mtDNA, forming a complex that is transported to the lysosome for degradation.	Modulates inflammation by targeting mtDNA removal and inhibiting the STING pathway.	[804]
		The development of BB‐Cl‐bimidine, LB244, has shown nanomolar potency and significantly enhanced selectivity across the entire prote.	This new chemical entity is able to directly inhibit the signaling of STING.	[805]
		Astin C was screened from the cyclic peptide library, which can competitively to the C‐terminal ligand‐binding domain of STING, blocking the recruitment of IRF3 to the STING signaling complex.	Inhibiting the activation of downstream signaling pathway mediated by STING.	[806]
		Gelsevirine promoted STING K48‐linked polyubiquitination and MG‐132.	Reversal of gelseverines inhibition of IL‐1β induced STING/TBK1 pathway activation in chondrocytes.	[807]
		Mutant STING resulted in increased phosphorylation of STAT1, and Janus kinase inhibitor reduced constitutive up‐regulation of phosphorylated STAT 1 in patient lymphocytes.	Interferon production is dependent on STING, and JAK inhibitors block IFNB1 transcription in vitro and continuously stimulate interferon signalling.	[808]
		SURF 4 is an articulator molecule, COPA maintains immune homeostasis by regulating STING transport in the Golgi, activated STING contributes to immune dysregulation in COPA syndrome.	COPA is a subunit of COPI that mediates Golgi transport to the endoplasmic reticulum, defective COPI transport leads to ligand‐independent activation of STING.	[809]
		Inhibition of palmitoylation of STING and elimination of the type I interferon response.	Introduction of 2‐BP or Cys 88/91 Ser mutations effectively suppresses the response induced by STING variants.	[810]
		The STING antagonist SN‐011 locks STING in an open inactive conformation.	It inhibits the induction of interferon and inflammatory cytokines activated by cGAS‐STING overexpression or SAVI STING mutants.	[787]
		The STING inhibitor MRT67307 significantly enhances the expression of exogenous proteins even 24 hours after their removal.	The STING pathway is activated by exogenous DNA in the cytoplasm, blocking exogenous gene expression and inducing DNA degradation.	[811]
		Single systemic administration of cAIMP mobilises and activates the human innate response through STING signalling in a chimeric level‐dependent manner.	The cGAMP analogue cAIMP elicits a STING‐dependent human IFN response in humanised NOG mice	[812]
		Two CMA molecules bind to the central cyclic di‐guanosine monophosphate binding pocket of the STING dimer and fold the cap region in a manner similar to, but partially different from, c‐diGMP.	CMA is a potent type I interferon inducer; CMA binds directly to STING and triggers a potent antiviral response via the TBK 1/IRF 3 pathway.	[813]
		diABZI induces activation and dimerisation of STING, followed by TBK 1/IRF 3 phosphorylation, leading to a type I IFN response.	diABZI is a STING agonist, internalised into the cytoplasm via an unknown receptor.	[814]
		It balanced the cGAS‐STING axis in order to enhance the therapeutic effect of stroke by synergistically combining the DNA mimetic enzyme Ce4^+^ enzyme and STING inhibitor.	Combining the anti‐inflammatory strategies of STING inhibition and dsDNA elimination, a C‐176‐loaded DNA mimetic enzyme artificial enzyme was constructed.	[815]
	Blocking the AIM2 pathway	NET is directly phagocytosed by macrophages and then stimulates AIM 2 inflammasome‐dependent macrophage pyroptosis in vitro.	The AIM 2 inflammasome activation inhibitor ODN A151 attenuates the progression of chronic liver inflammation/fibrosis in vivo.	[788]
		In patients with AKI, protein expression of the AIM 2 complex is increased and activation of the CD 73 signalling pathway is also detected.	The protective effect of Roxadustat (FG‐4592) against AKI is mediated by induction of CD 73 and inhibition of AIM 2 inflammatory vesicles.	[816]
		Ac‐YVAD‐cmk inhibits the activation of caspase‐1 pathway 24 h after cerebral haemorrhage and reduces the release of mature IL‐1β/IL‐18 in the perihematoma of cerebral haemorrhage rats.	Ac‐YVAD‐cmk inhibits the caspase‐1 pathway and reduces inflammatory reactions in cerebral haemorrhage.	[817]
		NLRC 4 acts as a sensor of caspase‐1 activation and is involved in caspase‐1 activation in DN and glucose‐stressed HK‐2 cells.	Treatment of diabetic animals with VX‐765 inhibited inflammatory cell infiltration and expression of septicity‐associated proteins and blocked caspase‐1 immunoreactivity.	[818]
		The first phase of NLRP 3 inflammasome activation is in response to TLR activation or some cytokine receptors. VX‐765 inhibits NLRP 3 inflammasome to block the response.	VX‐765 inhibits elevated levels of ROS inducing NF‐κB gene transcription and subsequently mediates NLRP 3 triggering via the transcriptional pathway.	[819]
		Autoinflammatory diseases caused by mutations in IL‐1 R1 disrupt the negative regulation of IL‐1 Ra and lead to unopposed activation of the IL‐1 pathway.	Rilabnacept can act as an IL‐1 Trap that captures only IL‐1β and IL‐1α without IL‐1 Ra.	[820]
		The targeted antioxidant Mito‐TEMPO scavenges mitochondrial reactive oxygen species.	Reverses calpain‐1‐mediated NLRP 3 inflammatory activation and cell death.	[821]
	RAGE inhibitors	Melanocytes treated with the JAK inhibitor ruxolitinib reduced HMGB1 and MX1 expression as well as activation of JAK1 and signal transducer and activator of transcription 1.	Small hairpin RAGE‐infected cells showed reduced response to human recombinant HMGB 1 and decreased MX 1 expression and JAK activation.	[822]
		The small molecule glycosylation end‐product receptor inhibitor TTP 488/Azeliragon interferes with advanced glycosylation end‐product receptor signalling.	Anti‐tumour inhibitor TTP 488 (Azeliragon) inhibits metastasis in triple‐negative breast cancer.	[823]
		Huang‐Lian‐Jie‐Du decoction attenuates inflammation and focal death by modulating the metabolic profile of carbonyl compounds and reducing carbonyl stress.	Huang‐Lian‐Jie‐Du decoction inhibits AGEs/NF‐κB pathway and exerts beneficial effects on diabetic encephalopathy.	[824]
Disrupting downstream signaling pathways	NF‐kB inhibitors	Bortezomib inhibits the activity of the core subunit β5i in the immunoproteasome and reduces the binding of β5i to NLRP 3.	Bortezomib inhibits NLRP 3 inflammasome activation and NF‐κB pathway.	[825]
		Wood flavonoids and/or salazosulfapyridine, both treatments reduced oxidative stress, impeded apoptosis and reduced immunohistochemical expression of caspase‐3.	Wood flavonoids and/or salazosulfapyridine, both of which reduced the upregulation of the INF‐γ/JAK 1/STAT 1 and INF‐γ /TLR‐4/NF‐κB signalling pathways.	[826]
		Aspirin inhibits RANKL‐induced osteoclast differentiation of dendritic cells by suppressing NF‐κB and NFATc 1 activation.	Anti‐inflammatory drugs that inhibit NF‐κB by blocking the degradation of IκBα.	[827]
		Sodium salicylate significantly reverses exercise‐triggered activation of NF‐κB signalling in mouse gastrocnemius muscle.	Anti‐inflammatory drugs that block phosphorylation and degradation of IκBα and inhibit NF‐κB.	[828]
		Inhibition of the HIF‐1α/NF‐κB cascade to regulate ROS clearance and macrophage repolarisation.	Glucocorticoid receptor agonist, inhibits NF‐κB activation.	[829]
		BAY 11–7082 antagonises I‐κB kinase‐β and prevents nuclear translocation of NF‐κB; it also inhibits the NOD‐like receptor family, NLRP 3 inflammasome activation.	Inhibitor of IκBα phosphorylation and NF‐κB, selectively and irreversibly inhibits TNF‐α‐induced IκB‐α phosphorylation and reduces the expression of NF‐κB and adhesion molecules.	[830]
		Resveratrol has significant anti‐inflammatory effects and inhibits HIF‐1α‐mediated angiogenesis via the TLR 4/NF‐κB signalling pathway.	Natural polyphenols with antioxidant, anti‐inflammatory, cardioprotective and anticancer properties, with a wide range of targets including NF‐κB, JAK, etc.	[831]
	JAK/STAT inhibitors	Analysis of synovial tissues from RA patients revealed pathogenic cells in the effusion that alter gene expression, molecular pathway changes such as JAK/STAT.	Tofacitinib reversibly inhibits JAK1 and JAK3 and, to a lesser extent, JAK2 and TYK2 in vitro.	[832]
		Upadacitinib is an oral selective Janus kinase inhibitor being investigated for the treatment of Crohn's disease.	Upadacitinib is considered a highly selective JAK1 inhibitor.	[833]
		JAK inhibition suppresses activation of cytokine signalling pathways involved in inflammation and joint destruction in rheumatoid arthritis.	Peficitinib is a pan‐JAK inhibitor that irreversibly binds to JAK1, JAK2, JAK3 and TYK2.	[834]

Abbreviations: AIC, abdominal infectious complication; AIM, absent in melanoma; AKI, acute kidney injury; ALA, alpha linolenic acid; Bax, bcl‐2‐associated x protein; BBB, blood‐brain barrier; CIRI, cerebral ischemia‐reperfusion injury; cGAS, cyclic GMP‐AMP synthase; CMA, 10‐Carboxymethyl‐9‐acridinone; COPI, coat protein complex I; DDP, cisplatin; diABZI, diamidobenzimidazole; DIDS, 4,4'diisothiocyanate−2, 2'−disulfonic acid, DNase, deoxyribonuclease I; I/R, ischemia/ reperfusion; LPS, lipopolysaccharide; MHT, menopausal hormone therapy; MICL, myeloid inhibitory C‐type lectin‐like; miMOMP, minority mitochondrial outer membrane permeabilization; NETs, neutrophil extracellular traps; NEs, neutrophils; NF‐κB, nuclear factor κB; NLRP3, NOD‐, LRR‐, and pyrin domain‐containing protein 3; PAD4, peptidylarginine deiminase 4; PROTAC, proteolysis‐targeting chimeras; PRT, post‐renal transplantation; STAT1, signal transducer and activator of transcription 1; TA, tartaric acid; TREX1, three prime repair exonuclease 1; VDAC1, oltage‐dependent anion channel protein 1; ZBP1, z‐DNA binding protein 1; 2‐BP, 2‐bromopalmitate.

With a deep understanding of the complex innate immune responses and downstream inflammatory pathways of cfDNA, addtional strategies for inflammation modulation have emerged [375, 376, 377]. For example, inhibiting the recognition site where cfDNA activates cGAS, altering the spatial conformation of cfDNA complexes to prevent matching the TLR9 lumen, and regulating the functions of innate and adaptive immune effector cell [378, 379]. Furthermore, cfDNA can form complexes with endogenous proteins or peptides, which protect it from degradation and promote its internalization by immune cells, thereby further activating TLRs. Endogenous proteins or peptides, such as LL37, HMGB1, HBD3, and IL‐26 have become new targets and orientations for inhibiting the activation of TLR9 [380, 381]. In addition, the development of medicine has witnessed the discovery of numerous new targets and the birth of related inhibitors for inflammatory regulation. For example, the recent studies have identified alanine‐tRNA synthetase 1/2 (AARS1/2) as L‐lactic acid sensors that modify cGAS at lysine site through ATP‐dependent lactylation, thereby inhibiting its DNA recognition and cGAMP synthesis, thereby suppressing the innate immune pathway [382].

### 4.2 Inhibitors of cfDNA Generation

Multiple inhibitors that reduce cell apoptosis, preventing cell necrosis and inhibiting cell active secretion can be developed to suppress the production of cfDNA and provide novel ideas for the treatment of inflammatory diseases [383, 384, 385, 386, 387] (Figure 3). First, drugs can be designed targeting cell death pathways [388, 389, 390, 391, 392, 393]. Apoptosis, as the main source of cfDNA, can be reduced by regulating intracellular apoptosis signaling pathways by targeting key molecules, including the caspase family, B‐cell lymphoma‐2 (Bcl‐2) family, tumor protein 53 (p53), and death receptor pathway, etc [394, 395, 396, 397, 398, 399, 400]. For example, Zhou et al. [377] developed a specific cfDNA binding agent G3‐8, containing high‐density helical peptide molecular fragments to block apoptotic signals in pathological states (e.g., systemic lupus erythematosus), and reduce the DNA release from apoptotic bodies. Targeting the regulation of endogenous apoptotic pathways associated with mitochondria, Cyclosporin A and related agents stabilize mitochondrial membranes and reduce mtDNA release, while rapamycin (an mTOR inhibitor) and allantoin, clear damaged mitochondria and prevent mtDNA leakage [401, 402, 403, 404, 405, 406, 407, 408, 409, 410, 411, 412]. Victorelli et al. [401] used the small molecule BCL‐2‐associated X protein (BAX) inhibitor BAI1 to suppress the conformational changes during BAX activation, preventing mitochondrial translocation and oligomerization of BAX, and thus exerting the effect of inhibiting mtDNA release and BAX activation. Besides, the application of necroptosis inhibitors, such as RIPK1/3 inhibitors (e.g., Necrostatin‐1 or GSK872), can also block the necrotic signaling pathway and reduce DNA leakage caused by cell membrane breakdown [413, 414, 415, 416, 417, 418, 419, 420].

Cells can also actively secrete DNA into the extracelluar space. Interventions targeting inhibiting the active secretory processes and/or regulating the cytokines and signaling pathways involved in active secretion may be helpful. For example, inhibition of the TGF‐β signaling pathway may reduce the active secretion of cfDNA [421, 422, 423]. The use of GW4869 (neutral sphingosines inhibitors) or targeting Rab GTPases can reduce the release of exosomes, while Rho kinase inhibitors (such as Y‐27632) can inhibit cytoskeletal remodeling, regulate microvascular formation, and reduce microvascular release [424]. Liu et al. [425] proved that pyrin inflammatory vesicles drive NET‐induced macrophage pyroptosis, and alpha‐linolenic acid may alleviate lung injury by inhibiting the activation of pyridine inflammasome‐driven macrophage pyroptosis. Furthermore, the PAD4 inhibitors (Cl‐amidine, GSK484) and neutrophil elastase inhibitors (Sivelestat Sodium) can inhibit the formation of NETs from the origin and have been approved to treat inflammatory diseases [426, 427, 428, 429, 430, 431, 432, 433, 434].

Although eliminating cfDNA has the potential to treat inflammatory diseases, the relevant research is still in its infancy. No related chemical or biological drugs specifically inhibiting cfDNA production have entered clinical trials. Inspired by pyrrolimazole polyamide (PIP), which is a DNA groove binding agent composed of N‐methylpyrrole and N‐methylimidazole components [435, 436, 437, 438, 439], PIP conjugates can be designed and constructed to achieve sequence‐specific alkylation of target adenine by introducing other functional groups, such as PIP mitochondrial penetration peptide conjugates and PIP alkylating reagents. These conjugates alter heterogeneity of cfDNA in living cells, and provide important insights for the development of chemical drugs [440, 441]. Besides, peptide‐structured nanocavenger is synthesized through self‐assembly strategies, can target the clearance of mtDNA from cells, inhibit the STING pathway, and regulate inflammation [442, 443].

Besides, promoting mitophagy, inhibiting mitochondrial permeabilization, preventing mtDNA release, degrading extracellular/circulating mtDNA, and blocking mtDNA‐induced inflammatory signaling have gradually become new anti‐inflammatory strategies on a laboratory scale [444, 445, 446, 447, 448, 449, 450]. Drug discovery strategies targeting the regulation of the disease of microenvironment and inflammatory pathways can be accomplished through nutritional supports, modulation of cellular metabolism, and interference with the packaging and secretion mechanism of DNA, providing foundational references and important supports for cfDNA intervention [451, 452, 453, 454, 455]. Additionally, circulating cfDNA levels can be indirectly reduced by enhancing macrophage clearance to accelerate apoptotic cell clearance. For example, Medzhitov et al. [456] demonstrated that IL‐10 plays a pivotal role in suppressing glycolytic dysfunction and eliminating ROS‐generating mitochondria through regulating LPS‐induced glycolytic conversion in macrophages.

In conclusion, the drug discovery based on reducing apoptosis, preventing necrosis, inhibiting active secretion of cfDNA, and achieving mitochondrial metabolic reprogramming will provide new approaches for the treatment of inflammatory diseases.

### 4.3 cfDNA Degrading Enzymes

Extracellular DNases are crucial for the degradation of cfDNA. Based on their spatial distribution, these DNases can be divided into DNase I and DNase‐I‐like 3 (DNaseIL3), located in the extracellular space, DNase II, phospholipase D3 (PLD3), and phospholipase D4 (PLD4), found in the phagolysosome, and TREX1, also known as DNase III, localized at the endoplasmic reticulum [455].

DNase I is an endonuclease that can digest single or double‐stranded DNA. Given its efficacy in degrading cfDNA, recombinant DNase I (marketed as Tigerase and Pulmozyme) has been approved for clinical treatment of pulmonary cystic fibrosis [457]. Zhang et al. [458] established an extracorporealmembrane oxygenation (ECMO)‐supported sepsis rat model and evaluated the effects of DNase I on hepatic damage in sepsis, suggesting that DNase I can mitigate liver injury via suppressing neutrophil infiltration and inhibiting the NLRP3 signaling pathway. In another study, Xia et al. [459] reported that DNase I substantially suppressed NETs production, thereby promoting angiogenesis, enhancing motor function, and improving regional angiogenesis, limb perfusion and motor function, and improving limb perfusion following post‐ischemia reperfusion injury. To prolong the lung retention time of DNase I and improve the clearance efficiency of cfDNA, Huang et al. [460] cross‐linked DNase I with albumin using glutaraldehyde to prepare a DNase I nanomotomotor system (DNase‐I/HSA NMs), which showed excellent enzymatic activity, physiological stability, and biosafety, and had enhanced degradation efficiency on cfDNA. Furthermore, DNase I has been explored for NETs disruption in experimental models of diabetic wounds treatment in a previous study [461, 462]. In order to extend the half‐life of DNase I, Wang et al. [463] combined DNase I with oxidized hyaluronic acid via Schiff base reaction for efficient NETs disruption in RA treatment. Furthermore, DNase IL3 digests cfDNA released from apoptotic cells into extracellular spaces. DNase II, located within lysosomes, functions by clearing dsDNA produced during apoptosis and phagocytosis, whose deficiency significantly enhances IFN‐1 signaling. PLD3 and PLD4, in particular, possess single‐stranded 5’‐exonuclease activity, which is crucial for further degrading ssDNA to suppress TLR9 or cGAS activation [464, 465, 466, 467].

TREX1, is the main 3'‐5' restriction exonuclease in the cytoplasm, expressed in most tissues and cell types of mammals. It is a powerful DNA‐degrading enzyme in the cytoplasm that can rapidly degrade both double‐stranded and single‐stranded DNA and has recently been shown to inhibit the innate immune response in cells [468]. TREX1 maintains the immune tolerance by degrading DNA in the cytoplasmic to prevent abnormal inflammation and autoimmune responses. Studies have found that adeno‐associated virus (AAV)‐mediated local overexpression of TREX1 significantly reversed the accumulation of cfDNA, inhibited the release of inflammatory cytokines, and improved synovial tissue damage in RA rats, while knockout of TREX1 increased cfDNA levels and aggravated the activation of abnormal immune responses [159]. Besides, Yu`s group found that inhibition TF rapidly triggered the degradation of TREX1 could be rapidly triggered, thereby restoring micronucleus accumulation and improving the phosphorylation and functional activity of the STING/TBK1 cascade by degrading cfDNA [468].

Specifically, endonuclease G (EndoG) is the most abundant and active mitochondrial endonuclease in mammals, mainly located in the mitochondrial intermembrane space, and plays important roles in apoptosis, cell proliferation, DNA recombination, mitochondrial DNA synthesis, and oxidative stress responses [453, 469, 470, 471]. EndoG is involved in the degradation of damaged mtDNA, while cells depleted of EndoG release high levels of mtDNA outside the mitochondria without affecting total mtDNA content or inducing apoptosis [472]. In senescent cells, mitochondrial ROS (mtROS) induces mtDNA release and EndoG translocation into the nucleus, thereby preventing mtDNA degradation by sequestering this endonuclease [473, 474, 475]. Supplementing with exogenous EndoG facilitates mtDNA degradation and helps maintain cellular homeostasis.

Artificial DNA enzymes have also been developed. Liang et al. [473] developed a series of polyimidazole‐based artificial DNases with various modifications, which were prepared by reversible addition‐fragmentation chain transfer (RAFT) copolymerization using triethylene glycol acrylate and imidazole‐modified methacrylamide as monomers. The imidazole residue was identified as the key site for catalyzing cfDNA degradation. Experiments revealed that the imidazole‐containing polymers were able to hydrolyze the phosphodiester bond and degrade cfDNA, which could alleviate RA and Acardi‐Goutières syndrome caused by DNase deficiency in rats. Drug discovery based on the local overexpression of extracellular DNase or artificial DNase aims to inhibit inflammation and treat inflammatory diseases by cfDNA degradation.

### 4.4 cfDNA Scavenging Agents

**FIGURE 7:**
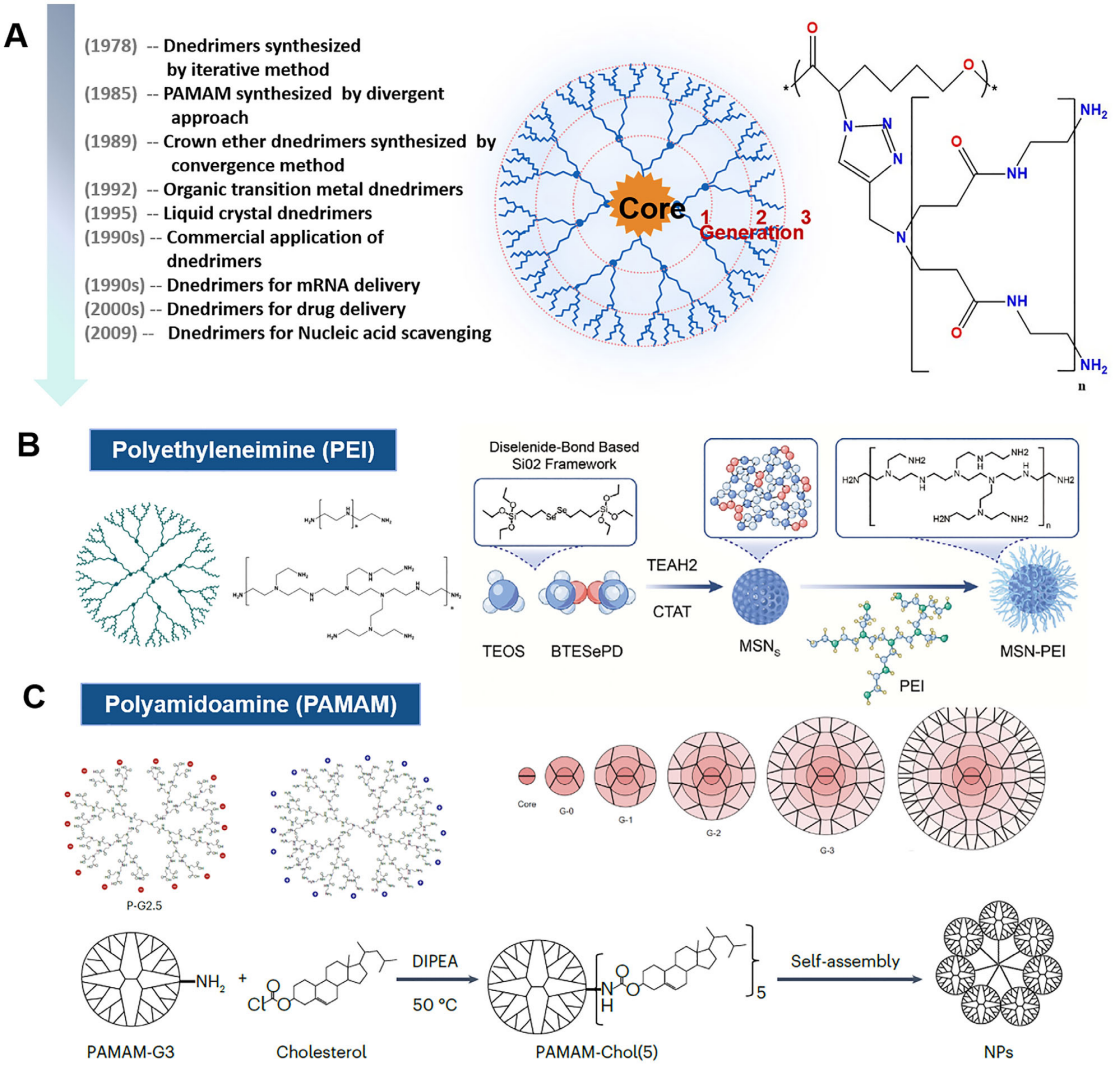
Dendrimers for cfDNA removal. (A) Timeline diagram of milestone discoveries in dendritic polymers and the structure of dendrimers. Figure was created with BioRender.com. (B) Schematic structure of PEI and synthesis process of MSN‐PEI nanoparticles [481]. Copyright 2024 John Wiley and Sons. (C) Schematic structure and modification strategy of PAMAM‐G3 [482].Copyright 2022 Springer Nature.

The removal of cfDNA has emerged as a hotspot in inflammation regulation, in which positively charged polymers and polymer‐modified nanomedicines display significant potential and efficiency. The development and application of dendritic cationic polymers, including PAMAM, PEI, PVP, and PL., which are widely used in drug delivery and nucleic acid therapy, have attracted attention in this field [476, 477, 478, 479, 480] (Figure 7A). Notably, to reduce toxicity and improve efficiency, researchers modify, dope, conjugate, or combine the above cationic polymers onto the surface of nanomaterials to construct polymer‐conjugated nanomedicines, uncovering the potential for controlled release, precise targeting, and multi‐functionality therapies.

#### 4.4.1 PEI‐Derived Cationic Polymers

Currently, a PEI polymer with the highest cationic charge density is the most widely used gene vector and transfection reagent. It is divided into branched PEI and linear PEI according to the differences in synthetic processes [483, 484] (Figure 7B). Owing to its unique charge characteristics and easily modified chemical structure, PEI can bind to the negatively charged cell surface residues and enter the cell through endocytosis [485, 486]. The acid‐catalyzed polymerization of azacyclobutane produces a dense network of amino groups that can be protonated and wrap around the negatively charged DNA through electrostatic interaction to form stable complexes [487, 488]. As expected, a substantial number of studies have confirmed its ability to scavenge cfDNA. For example, the PEI and PEI modified mesoporous silica NPs (PEI‐MSN) exhibited high cfDNA binding affinity, remarkable suppression of TLRs signaling, and distinct relieving of oxidative stress, displaying therapeutic potential in various inflammatory diseases, including IBD, sepsis, and refractory muscle fibrosis [157, 160, 489].

In terms of transfection efficiency, the cfDNA scavenging capacity of PEI on cfDNA is influenced by multiple factors, such as molecular weight, charge density, modification strategy, and cell type [483, 484, 490, 491]. In Hela and L929 cells, 25kDa PEI showed the best transfection efficiency, indicating that PEI with a larger molecular weight has stronger DNA‐binding ability and cell membrane penetration [485, 492, 493, 494]. When used for cfDNA scavenging, 25kDa MSN‐PEI and 25kDa PEI with high molecular weight both exhibited better cfDNA binding ability and anti‐inflammatory effects than 800Da MSN‐PEI and 800 Da PEI, respectively. The deacetylation degree during the synthesis of linear PEI affects its physicochemical and biological properties [486, 494, 495, 496, 497]. Compared to commercial PEI, the fully deacetylated linear PEI showed 10 000 times higher capacity of delivering DNA to the lungs. Both PEI 25 and PEI 87 can load siRNA for gene therapy of lung diseases, with inhibition rates of 77% and 93%, respectively [498, 499, 500].

Charge density is another critical factor, where branched PEI with a higher charge density binds more DNA and forms more stable complexes, thus improving binding efficiency [501, 502, 503]. It is worth mentioning that PEI with higher molecular weight and charge density shows stronger DNA binding and cell membrane penetration abilities, but also greater cytotoxicity [487, 504, 505]. It is required to balance cfDNA scavenging efficiency and cytotoxicity by flexibly adjusting the molecular weight, structure, and assembly of PEI when constructing nanomedicines [506, 507]. To reduce side effects, multiple strategies, such as decreasing dose and molecular weight, introducing degradable disulfide bonds, and conjugating with cyclodextrin have been adopted [508, 509]. Meanwhile, by altering the surface state of PEI, such as application of branched architecture, introduction of functional groups (e.g. glycosylation, acetylation, pegylation, thioketones [TK] grafting, polypeptide modification, and targeted miety conjugation), and/or other intelligent components, nanomedicines with low cytotoxicity, high safety, good accuracy, and targeted delivery can be achieved, providing new ideas for constructing efficient and safe nanomedicines [510, 511].

#### 4.4.2 PAMAM‐Derived Cationic Polymers

PAMAM, another polymer commercialized in 1990, possesses a nanoscale size, radial dendritic structure, and high hydrophilicity. It consists of an ethylenediamine core, repeating branched methacrylate and ethylenediamine units radially attached to the core, and the terminal groups on the branches [512, 513, 514] (Figure 7C). It is divided into the first to fifth generations according to the number of repeated branching units, with the number of functional groups on the PAMAM surface increasing exponentially [515, 516]. Owing to its huge internal cavity, large specific surface area, and good solubility, PAMAM is capable of forming stable colloidal solutions in the aqueous phase, and exerts multiple physical effects, such as electrostatic interactions, hydrophobic action, and chemical bonding to deliver drugs [517, 518, 519]. It can interact with a variety of endogenous biomolecules in tissues as well as cells, thus enabling the delivery of therapeutic agents in vivo [520, 521].

Structurally, PAMAM carries abundant positively charged amine groups that interact with negatively charged DNA to form complexes [522, 523]. Growing evidence has disclosed its ability to scavenge cfDNA, where PAMAM (especially PG3) effectively removes cfDNA, inhibits TLRs activation, and prevents the inflammatory cascades in periodontitis, sepsis, and obesity [524, 525, 526, 527, 528].To prevent the possible dissociation of PAMAM micelles in vivo, Peng et al. [529] designed a library of biodegradable cationic polymers with precisely tuned charge density and molecular weight by adjusting the degree of polymerization and the generation of primary amine‐type PAMAM. The precise structure of PAMAM was designed and optimized with DNA binding, toxicity, inflammatory inhibition, and in vivo distribution, enabling optimal performance in suppression of TLRs signaling, synovial hyperplasia, and bone destruction. PAMAM‐modified NPs targeting cfDNA removal have been developed for multifunctional anti‐inflammatory treatment. Huang et al. [165] synthesized cationic polyamide dendrimers (G3@SeHANs) for the treatment of periodontitis, where PAMAM modification effectively reduced the rate of selenium release and the biodegradation of SeHANs, optimizing the rate and time of drug release. Zhou et al. [377] incorporated high‐density helical peptide molecular clips into a PAMAM‐based dendritic cationic polymer to acquire efficient capture and stable clearance of cfDNA through electrostatic attraction, salt bridging, and volumetric spatial confinement of rigid α‐helices, and finally achieved immune suppression by clearing immune‐stimulating cfDNA rather than inhibiting TLR receptors. It also significantly enhanced the binding ability of the nanomedicine to cfDNA and improved its bioavailability in vivo.

#### 4.4.3 Polyamino Acid‐Based Cationic Polymers

**FIGURE 8:**
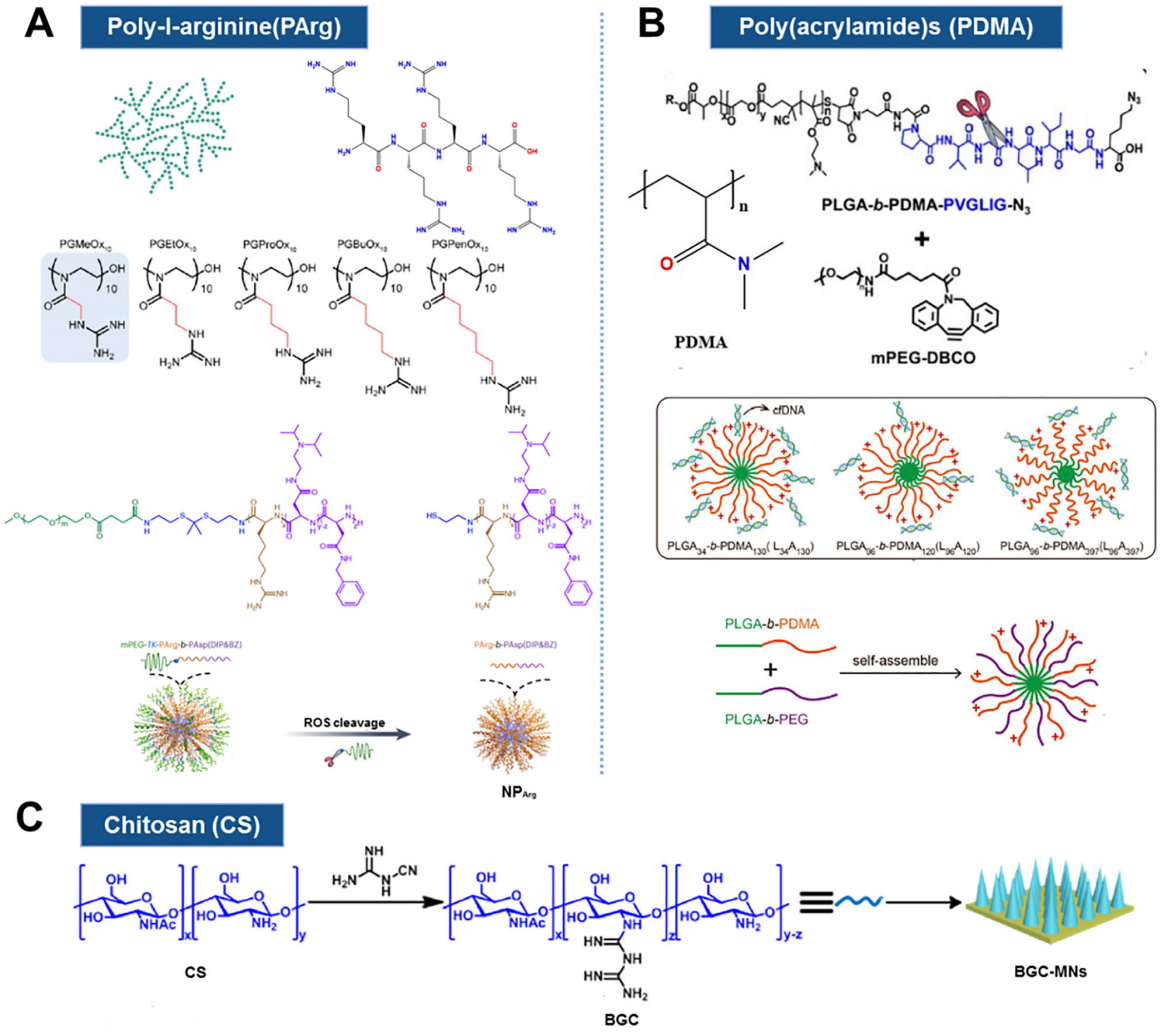
Cation polymers for cfDNA removal. (A) Structures of guanidinium functionalized poly (2‐oxazoline) s bearing different side chain spacer arms [530, 531]. Copyright 2022 Elsevier. Copyright 2023 American Chemical Society. (B) Structure of cationic nanoparticles formed by self‐assembly of PLGA‐b‐PDMA [530]. Copyright 2022 Elsevier. (C) Schematic structure of BGC‐MNs [532]. Copyright 2023 Elsevier.

Polyamino acids, including PLL and poly‐l‐arginine (P‐Arg), are early polymers used for nucleic acid delivery. They display high affinity, good biocompatibility, and favorable biodegradability. PLL is a peptide sequence composed of repeating lysine‐containing residues. By adjusting the synthesis conditions, chain lengths, and conformations of PLL, nanomedicines can be obtained to meet the biomedical requirements [533, 534, 535] (Figure 8A). Owing to its positive charge, PLL effectively removes cfDNA. It can also be combined with other polymers or NPs (e.g., polyethylene glycol [PEG], polylactic acid) to form composite nanomedicines. Wang et al. [158] applied this strategy for the treatment of RA (Figure 8A). With the assistance of PLL, the biocompatibility and targeting of the nanomedicine were dramatically improved, and meanwhile, after MMP‐mediated cleavage of PEG, positively charged PLL was exposed to scavenge cfDNA in the presence of specific enzymes.

Moreover, P‐Arg, exhibiting plenty of guanido groups, shows a high affinity to the cell membrane and nucleic acids. Inspired by the transcriptional activator (TAT) with abundant arginine residues, Chen's group developed a P‐Arg‐based nanomedicine, which effectively inhibited the inflammatory response caused by cfDNA through TLR9 [536]. Herein, the guanidine (Gua^+^) moieties of P‐Arg had strong cfDNA binding by forming a Gua^+^/PO_4_^−^ salt bridges.

#### 4.4.4 PVP‐Based Cationic Polymers

As a nontoxic and chemically stable polymer, PVP has excellent solubility, film formation, and biocompatibility, and is commonly used as a solubilizing agent or co‐solvent in pharmaceutical formulations [537, 538]. The molecular structure of PVP contains plenty of hydroxyl and amide groups, enabling further surface modification [539, 540]. The positive surface charge of PVP enables the purpose of scavenging cfDNA. For example, Li et al. [541] reported an NP (TMPP) containing tannic acid (TA), Mn, polymyxin B (PMB), and PVP for the treatment of sepsis. By coating PVP on the surface of TMPP, it alleviates inflammation, prevents the NPs aggregation, and maintains high monodispersity, which enhances its in vivo stability and functionality.

#### 4.4.5 Chitosan‐Based Cationic Polymers

Chitosan is a polysaccharide derived from the deacetylation of chitin, exhibiting good biocompatibility, non‐toxicity, and biodegradability. Its molecular structure is rich in amino and hydroxyl groups, endowing it with water solubility, structural tenability, and chemical modifiability [542, 543, 544]. In general, chitosan possesses excellent adsorption properties for a wide range of biomolecules [545, 546]. Specifically, the amino and hydroxyl groups on the surface of chitosan form hydrogen bonds with cfDNA, while the hydrophobic portion of chitosan helps to enhance the interaction with cfDNA [547, 548]. Via these forces, chitosan effectively captures cfDNA. Composition of chitosan with other materials (e.g., polyvinyl alcohol and gelatin) helps to form novel nanomedicines with multiple functions. For example, Liu et al. [532] prepared a cationic microneedle made of bisguanidine chitosan (BGC‐MNs), which was designed with a reference to the length, density, and arrangement of microneedles for effectively penetrating the skin (Figure 8C). Therefore, BGC‐MNs painlessly targeted the dermis and removed cfDNA to treat psoriasis.

#### 4.4.6 PDMA‐Based Cationic Polymers

For biomedical applications, PDMA can be synthesized in a flexible and versatile manner, and its physicochemical properties can be tailored by adjusting the degree of polymerization and the introduction of functional groups [549, 550, 551] (Figure 8B). The structure of PDMA, characterized by a cyclic polymer with an imide ring structure in its main chain, contains a variety of functional groups that enable it to form stable complexes with negatively charged cfDNA through electrostatic interactions and hydrogen bonding [552, 553, 554]. Recent studies have verified that PDMA‐modified NPs or hydrogels can effectively improve the clearance efficiency of cfDNA. Compared to PDMA, the nanomedicine (PLGA‐b‐PDMA) [530], formed by combining biodegradable poly (lactate‐glycolic acid) (PLGA) with PDMA, formed a stable complex with cfDNA through electrostatic interactions and thus achieved effective cfDNA clearance and potent inflammation blocking [555, 556] (Figure 8B). Given the capacity of PDMA in drug delivery, Liu et al. [530] developed a cationic nanomedicine for the treatment of RA using self‐assembled cNP‐pp‐PEG, with hydrophobic methotrexate (MTX) loaded into its core. It scavenged abnormally high levels of cfDNA and prevented RA inflammation.

#### 4.4.7 HDMBr‐Based Cationic Polymers

HDMBr is a cationic polymer famous for its ability to bind amicably to cells and tissues in living organisms, especially for its affinity to negatively charged biomolecules [557, 558]. HDMBr, with a large number of cationic groups, can form strong electrostatic interactions between the phosphoryl groups of cfDNA, prompting the formation of HDMBr‐DNA complexes [559, 560]. Subsequently, HDMBr facilitates the cellular uptake of cfDNA via endocytosis and achieves the degradation of cfDNA in cells. It also promotes cell repair and regeneration by regulating the intracellular environment, further enhancing the effect of cfDNA removal [561, 562]. These sequential mechanisms contribute to inhibiting the inflammatory cascade induced by cfDNA, thus demonstrating its potential in the treatment of cancer and inflammatory diseases [563, 564]. For example, HDMBr substantially reduced cfDNA levels in the tumor microenvironment, thereby inhibiting tumor cell proliferation and metastasis [557]. HDMBr is also applied in inflammatory diseases, such as RA and psoriasis, showing the potential to alleviate inflammation. Aswani et al. [557] used the HDMBr to neutralize mtDNA for the therapy of multiple organ dysfunction syndrome (MODS). In detail, HDMBr with a nanoscale size bound tightly to cfDNA and showed a noteworthy ability to concentrate DNA into nanocomplexes for cellular delivery.

### 4.5 cfDNA‐Mediated Inflammatory Signaling Inhibitors

With a deeper understanding of the complex innate immune responses and pathophysiological processes of cfDNA‐induced inflammation, other strategies gradually come forward. Blocking the cfDNA‐induced pro‐inflammatory signaling pathways (e.g., TLR9‐MyD88, cGAS‐STING, inflammasome AIM2, RAGE/NF‐κB, etc.) is considered as another effective strategy for anti‐inflammatory therapy [565, 566, 567, 568, 569, 570, 571, 572]. Commonly, these strategies include blocking TLR9 recognition of cfDNA CpG motifs, suppressing cytoplasmic cfDNA sensing, blocking RAGE‐cfDNA interaction, and suppressing NF‐κB activation, all of which work by targeting cfDNA recognition receptors [573, 574, 575, 576]. Inhibitors targeting multiple action points in the cGAS‐STING pathway, such as inhibiting the activity of cGAS (e.g., RU. 521, aspirin, PF‐06928215), preventing the synthesis of cGAMP, and blocking the activation of STING (e.g., H‐151, C‐176, and PROTAC), are also one of the current research hotspots [577, 578, 579, 580, 581, 582, 583]. Subsequently, disrupting the downstream signaling pathways, including inhibiting TBK1, IKK, TRIF, and IRF7, suppressing NF‐κB activation, and blocking IFN responses induced by cfDNA‐TLR9/RAGE or cfDNA‐STING signaling, merits attention [584, 585, 586, 587, 588]. Further details on the discoveries and applications of the substances inhibiting cfDNA‐related inflammatory signaling are shown in Table 2 and Figure 6.

## 5 Engineered Design on Nanomedicines for Targeted cfDNA Clearance

### 5.1 Drug Loading

**FIGURE 9:**
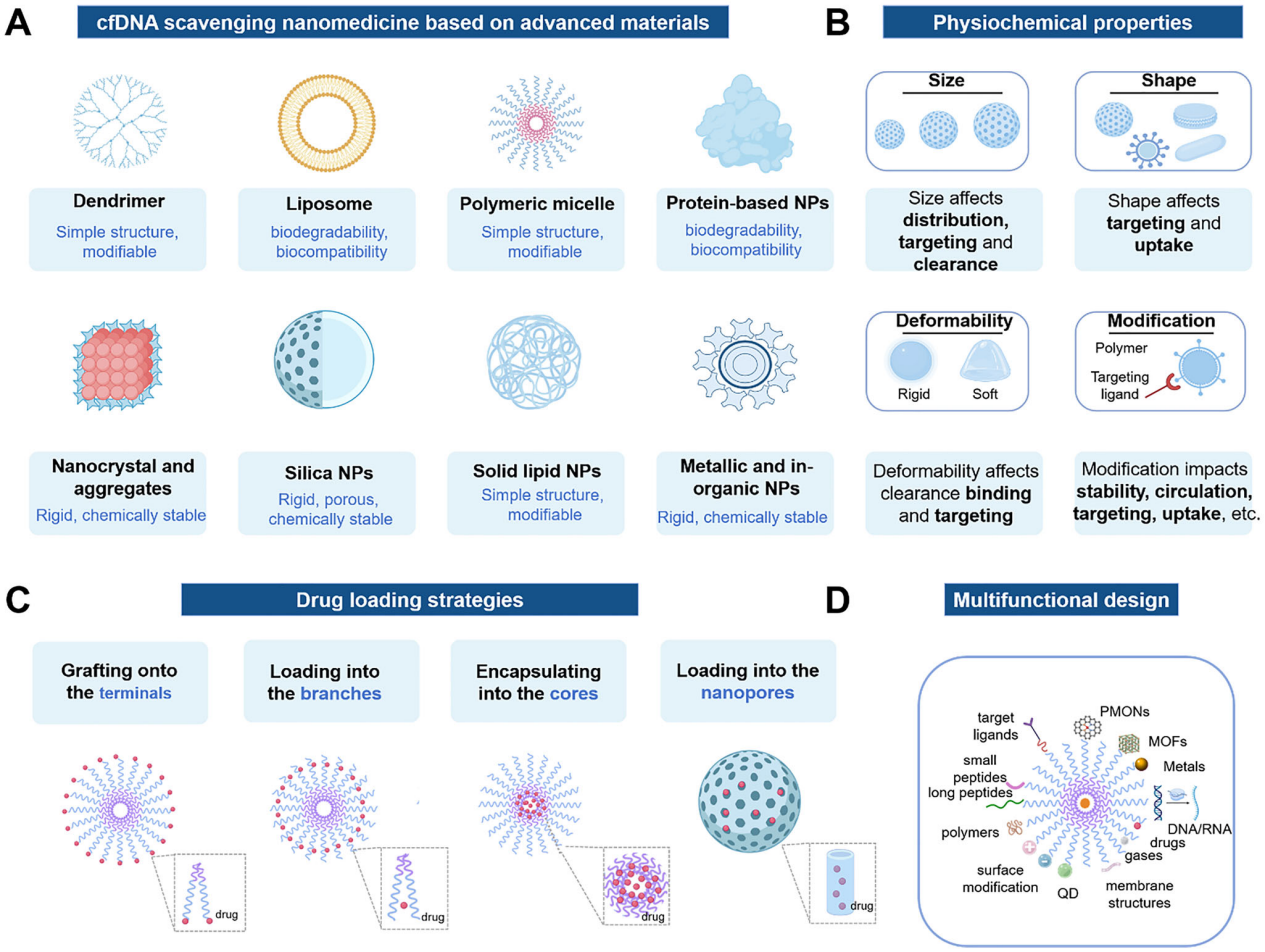
cfDNA‐scavenging nanomedicines. Examples of material compositions (A), the critical physicochemical properties including size, shape, deformability, and surface modification (B), drug loading strategies including grafting onto terminals, loading into the branches, encapsulating into the cores, and loading into the nanopores (C), and multifunctional design principles (D) for cfDNA‐scavenging nanomedicines. Figure was created with http://www.BioRender.com.

To our knowledge, numerous therapeutic agents designed for cfDNA scavenging exhibit nanoscale dimensions, extensive internal cavities, branched structures, and dense functional groups, can be classified as polymers or nanomedicines. By rationally engineering the composition, size, shape, surface chemistry, and texture properties of these nanomedicines, and by modifying functional groups, responsive moieties, and specific ligands, researchers have unlocked their potential as promising drug delivery systems with the multifunctions of controlled release, precise targeting, and microenvironment reprogramming [589, 590, 591, 592, 593] (Figure 9).

First, a variety of drugs, with anti‐inflammatory, immunoregulatory, and antibacterial activities, have been developed for inflammation manipulation [594, 595, 596, 597]. From a structural perspective, they can be divided into chemical drugs, protein/peptide drugs, gene drugs, and metal drugs [598, 599, 600, 601, 602, 603, 604]. Both internal and external drug loading strategies allow the incorporation of therapeutic agents for inflammatory diseases. External drug loading is usually achieved through electrostatic adsorption, covalent or non‐covalent connection on nanosystems [605, 606, 607, 608, 609, 610, 611]. In detail, molecules can chemically form solid covalent bonds with the functional groups onto the surface of polymers or nanomaterials, thereby regulating the physical states and dissolution properties of the drugs and enabling controlled drug loading and release [612, 613, 614, 615, 616]. Internal drug loading, in comparison, mainly relies on the branched, porous, and cavity structure of the nanosystems, where drugs can be efficiently encapsulated via hydrophobic interactions or hydrogen bonds [617, 618]. In addition to passive encapsulation, drugs can also be attached to the nanosystems as branching units by covalent coupling for more precise drug release [619, 620, 621, 622, 623]. For example, the chemical drug MTX is a clinical first‐line drug to alleviate the symptoms of RA, but its systemic administration often leads to severe adverse effects such as anaemia and leukopenia [530]. To address this problem, Liu et al. [530] applied an innovative approach to load MTX into the core of PDMA‐based NPs cNP‐pp‐PEG via hydrophobic interaction to form MTX@cNP‐pp‐PEG. Once exposed to the inflammatory environment, MTX@cNP‐pp‐PEG exhibited dual functions of cfDNA scavenging and MTX release, which significantly suppressed the inflammatory response triggered by macrophages. With the assistance of the innovative delivery system, the dose of MTX was dramatically reduced with prolonged treatment in alleviating the symptoms of RA model rats even after 4 days. Although these two drug‐loading strategies have extensive applications in pharmaceutical development, selecting appropriate drug‐loading methods in practice necessitates comprehensive consideration of multiple factors, including drug properties (e.g., molecular weight, solubility, and stability), therapeutic objectives, and carrier characteristics.

On the other hand, cationic polymers, including PAMAM, PEI, PVP, PLL, and PDMA, are routinely used as non‐viral nucleic acid carriers, which possess an inherent capacity to deliver gene drugs [624, 625, 626, 627, 628]. For example, Li et al. modified PEI onto the surface of polydopamine (PDA) to serve as a Klotho gene carrier to prevent acute kidney injury [629]. In another case, a TK‐linked fluorinated PEG (TKPF) was employed as an effective gene/drug carrier to load the apoptotic gene PUMA plasmid and the anti‐inflammatory active ingredient tripterine for RA therapy [630]. Furthermore, the nanosystems usually have a three‐dimensional spherical structure, enabling them to bind to biological agents as receptor mimics. Typically, highly branched NPs with a polycovalent structure enable multiple contacts with the cargo. These studies have demonstrated the prospect of cfDNA scavenging for a wide range of applications in the biomedical field.

### 5.2 Inflammation‐Responsive Drug Release

**FIGURE 10:**
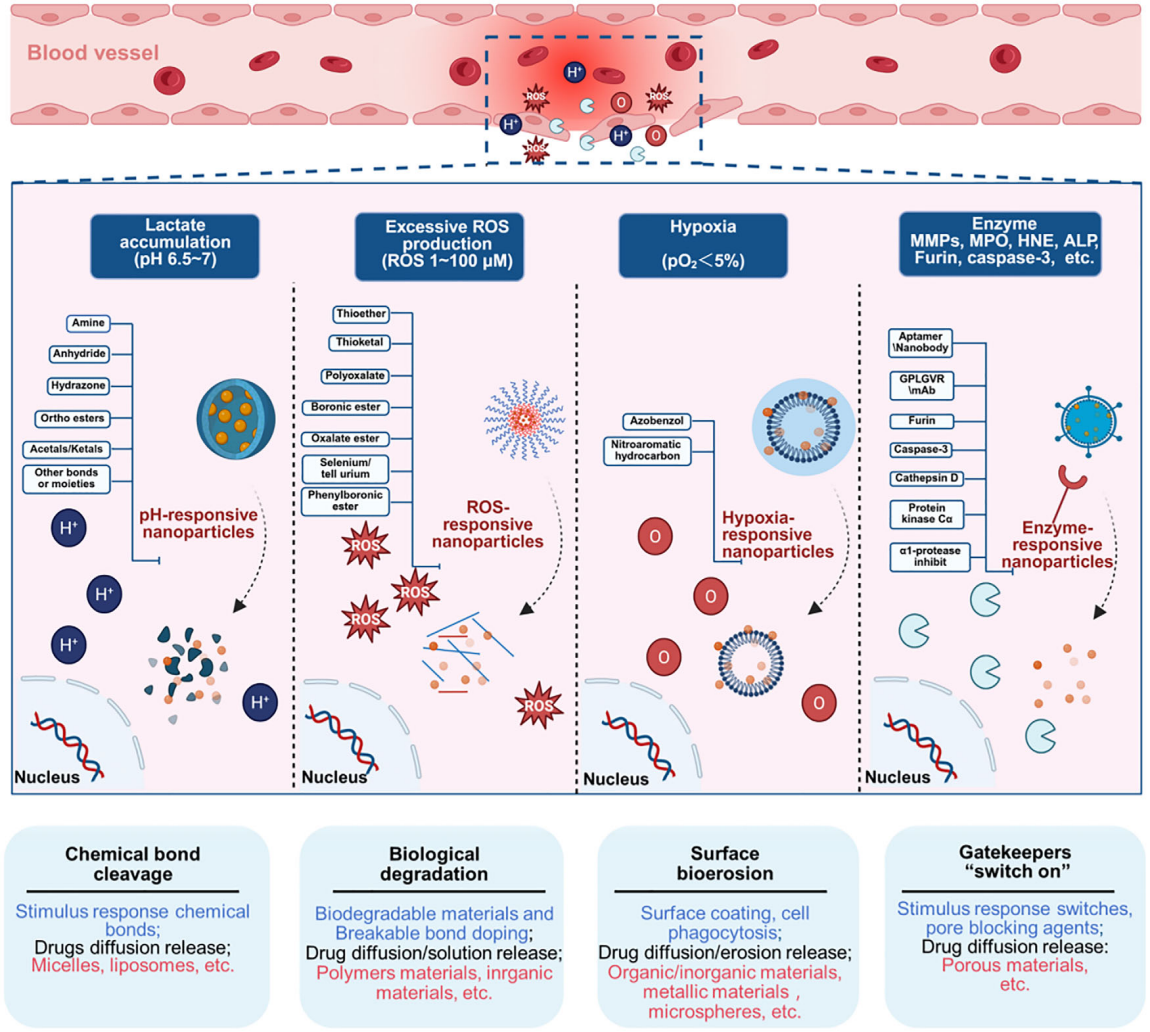
Inflammation‐responsive drug release of cfDNA‐scavenging nanomedicines. Responsive nanomedicines are designed to respond to various signals, including pH, ROS, hypoxia, and enzyme, by leveraging multiple release mechanisms such as chemical bond cleavage, biological degradation, surface bio‐erosion, and gatekeepers “switch on.” The corresponding functional moieties are indicated using blue borders. Figure was created with http://www.BioRender.com.

The coordination of a variety of immune or non‐immune cells and inflammatory mediators, including pro‐inflammatory enzymes, overproduced ROS, local acidification, hypoxia, and inflammatory mediators, constitutes an inflammatory microenvironment [631, 632, 633, 634, 635, 636, 637, 638] (Figure 10). Nanomedicines that respond to inflammatory microenvironments are triggered by various endogenous and/or exogenous biophysicochemical stimuli, which have gained increasing attention for improving therapeutic effects and reducing side effects [639, 640, 641, 642, 643, 644]. Especially, pH‐, ROS‐, and/or enzyme‐responsive functional nanosystems (with functional moieties highlighted in blue borders in the corresponding part of Figure 10) have been ingeniously designed for drug delivery in the therapy of inflammatory diseases [645, 646, 647, 648]. Generally speaking, the responsive mechanisms mainly involve the cleavage of chemical bonds, the degradation of materials, the erosion of surface coatings, and the break or activation of the gatekeepers or switches, which are the effective strategies for controlled drug release in a diffusion or erosion manner [649, 650, 651]. For example, Zhou et al. [652] constructed a charge reversal nanomedicine polyionic complex (PICsomes) with inflammation‐responsive cfDNA clearance and MTX delivery for autoimmune disease (AID) therapy. Under the acidic inflammatory microenvironment, PC underwent a charge reversal from negative to positive charge, and PICsomes degraded and released PEG‐PG and MTX, thus ameliorating the inflammatory microenvironment to promote tissue repair in the AID mouse model. Other than cfDNA, excessive ROS characterizes the microenvironment of inflammatory diseases, leading to a range of cellular injuries and inflammatory responses in pathological states. Liu et al. [536] developed a nanomedicine PEG‐TK‐NP_Arg_, which responded to ROS in the inflammatory environment to expose the functional cfDNA scavenging moiety, while avoiding adverse effects on normal tissues.

Plenty of inflammatory cells, such as neutrophils and macrophages, are present in inflammatory tissues, which secrete matrix metalloproteinases (MMPs), myeloperoxidase (MPO), neutrophil elastase, cathepsins, hyaluronidase, phospholipase A2 (PLA2), and cyclooxygenase (COX) [653, 654, 655]. Those inflammation‐associated enzymes are frequently upregulated at the initial stage of inflammatory diseases, which maintain abnormally high levels during the whole process of inflammation [656, 657, 658]. Among them, neutrophil elastase, cathepsins, and MMPs are the most commonly studied enzymatic triggers in inflamed sites for designing enzyme‐responsive nanomedicines [659, 660, 661]. For instance, cNP‐pp‐PEG was synthesized by linking to MMP2‐sensitive polypeptide (pp) [530]. Under physiological conditions, the surface cations of cNP‐pp‐PEG were shielded by the PEG shell. Once arriving at the inflammation sites, where MMP2 was overexpressed by abnormally activated fibroblasts, monocytes, and macrophages, the PEG shell was shed, exposing the cations for clearing cfDNA and inhibiting inflammation, thus improving the treatment accuracy and reducing the systemic toxicity.

Given the multiple biological triggers at inflamed sites, well‐designed multiple responsive delivery systems undoubtedly further improve the delivery efficiency. Zhang's group modified PLGA with different phenolic compounds, especially tyramine, which significantly enhanced the targeted aggregation of the nanomedicine at the site of inflammation, mainly owing to the fact that phenol groups can form free radicals under the action of local excess MPO and ROS in a pathological microenvironment [662]. It further formed polymers, promoting the in situ cross‐linking and tissue anchoring of the nanomedicine, showing therapeutic effects for IBD, acute liver injury, and acute lung injury [663, 664, 665, 666, 667].

### 5.3 Administration Routes and Targeted Drug Delivery

#### 5.3.1 Delivery Routes and Biodistribution

Based on the pathophysiological and anatomical characteristics of different inflammatory diseases, understanding the in vivo transport of drugs provides guidelines for the multifunctional design of therapeutic agents with higher specificity and fewer side effects [668, 669, 670, 671]. The regulation of the size, shape, structure, surface charge, surface modification, and delivery routes of the therapeutic agents via pharmaceutical strategies enables them to overcome multiple physiological barriers and achieve optimal targeting of different organs, cells, and molecules with improved absorption, targeting, and clearance properties [672, 673, 674]. Furthermore, after entering the body, therapeutic agents are likely to be recognized as foreign substances, cleared by the reticuloendothelial system (RES), and passively targeted to the liver and spleen, offering a convenient route for the treatment of liver diseases. For example, Kanasty et al. [675] modified the amino groups of PEIs with ethyl trifluoroacetate or perfluorobutyryl chloride to obtain PEI fluoride, which reduced the cytotoxicity of PEI, and significantly improved the delivery of siRNA in the liver. Continuous endothelial barriers further scatter the distribution of drugs, and contribute to their poor and slow accumulation into the target tissues [676]. Compared to naked G4 PAMAM (4 nm) and G5 PAMAM (5 nm) which are adsorbed by RES (mainly distribute in liver and spleen), G4 and G5 conjugated with antibody fragment (mAPP2 Fab) of lung fossa could efficiently cross the endothelial cells via a caveolae pumping system and rapidly accumulated in the lung at 10 and 30 min post injection, respectively [676]. Inhalation delivery can be applied to specifically deliver drugs into the lungs. Besides, the extracellular matrix of adipocytes is mainly composed of collagen and negatively charged biological macromolecules glycosaminoglycans (GAGs) [677, 678, 679]. The cationic PAMAM (P‐G3) could target the negatively charged extracellular matrix of visceral fat for the treatment of associated diseases [680]. Lipophilic P‐G3‐Chol NPs after cholesterol modification further improved the targeting and therapeutic ability [482].

The delivery route determines the absorption and biological fate of various therapeutic agents. Medicines can be delivered into the body through various routes, such as oral administration, intravenous, subcutaneous or muscular injection, mucosal administration, and local application [681, 682, 683, 684, 685, 686, 687]. Oral administration with the highest safety and compliance can exert a systemic therapeutic effect after being absorbed via the small intestine. It also allows drugs to directly act on the inflammatory tissue of IBD [688]. Besides, novel strategies are put forward to improve its ability to penetrate the mucus barrier. Shen et al. [689] embedded Mg in the PLAG@PEI shell to construct a microrobot, which achieved efficient autonomous movement and penetrated the intestinal mucus barrier by reacting Mg with water to produce hydrogen. For sepsis therapy [160], an intravenous administration directly delivers drugs into the circulatory system, with rapid therapeutic effect and high bioavailability. Specifically, topical therapy can directly act on the inflammatory tissues, improving the targeting ability and reducing systemic adverse effects caused by oral or injection administration. For instance, rectal administration can be used for the treatment of IBD [157], and inhalation delivery is a nice choice for lung disease. RA therapy is usually realized by injecting into the joint cavity, while drugs for periodontitis and skin inflammation can function by local injection, topical application, or microneedle [158].

#### 5.3.2 Targeted Strategies

**FIGURE 11:**
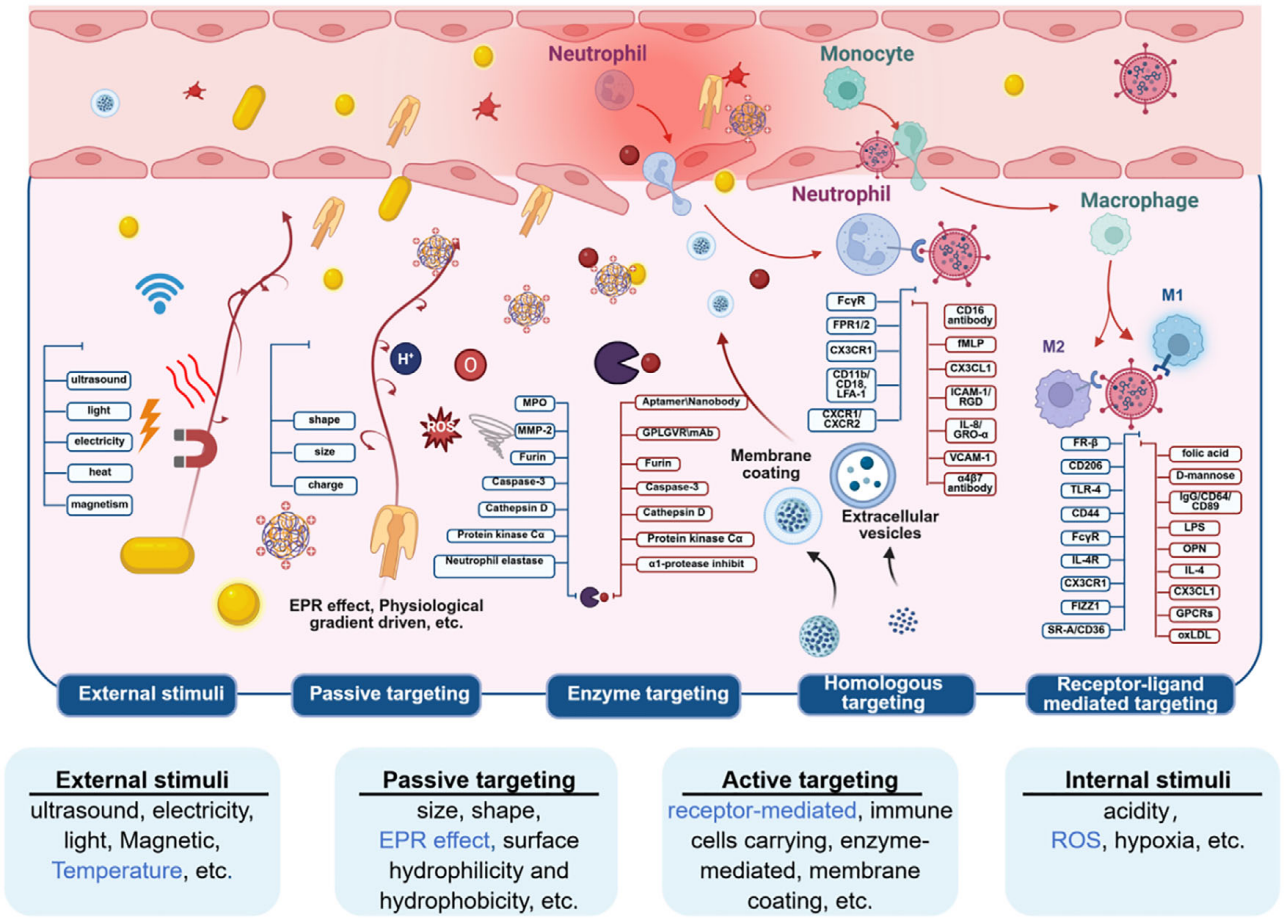
Targeted strategies of cfDNA scavenging nanomedicines. External stimuli, passive targeting, active targeting, and endogenous targeting can be used as combined tools for the multifunctional design of cfDNA targeting scavenging nanomedicines. The corresponding stimulus and functional moieties are indicated using blue borders and red borders, respectively. Figure was created with http://www.BioRender.com.

Target design of therapeutic agents should also focus on the enhanced vasculature permeability and biomedical clues in the inflammatory microenvironment. In practical exploration, external stimuli, passive targeting, active targeting, and endogenous targeting can be combined in a design to strike a balance between targeting ability, therapeutic effect, and toxicity (Figure 11). Passive targeting of nanomedicines can be significantly improved by precisely regulating the physicochemical properties (e.g., shape, size, surface charge) of cfDNA‐scavenging nanomedicines. Furthermore, enhancing the permeability and retention (EPR) effect produced by the changes of blood vessels and blood flow in inflammatory sites can further optimize their therapeutic outcomes [690, 691, 692, 693]. Plenty of inflammatory cells, such as neutrophils and macrophages, migrate to the inflammatory sites, eliminate pathogens, and release cytokines and enzymes, which are crucial for the treatment of numerous inflammatory diseases [694, 695, 696]. Especially, enzyme mediated and/or receptor ligand mediated active targeting strategies such as surface modifcation with dextran, D‐mannose, folic acid, oxidized low‐density lipoprotein (oxLDL) and MMP‐2 substrate peptide (GPLGVR), etc. (Some stimulus and functional moieties are highlighted in blue borders in the corresponding part of Figure 11), help to increase the uptake of nanomedicines by macrophages or neutrophils [697, 698, 699, 700, 701] also enhance homologous targeting inflammatory tissues. Except for acting as therapeutic targets, these inflammatory cells can engulf different nanomedicines, followed by migration into inflammatory sites, making them interesting “Trojan Horses” for targeted delivery into inflamed sites [702, 703, 704]. For example, macrophages tended to take up nanorods rather than nanospheres [705]. In another case, Luo et al. [706] designed a nanomedicine by free‐riding after being phagocytosed by neutrophils. Targeted design of nanomedicines can eliminate cfDNA or intervene in cfDNA‐related inflammatory pathways, which provide new approaches for the diagnosis and treatment of inflammatory diseases by using cfDNA as a biomarker or therapeutic target.

### 5.4 Inflammatory Microenvironment Reprogramming

Regulation of cfDNA‐related inflammatory signals requires multifaceted interventions targeting cells, molecules, and metabolic components. Therapeutic strategies need to be well designed to maintain immune homeostasis through suppression of pro‐inflammatory factors, enhancement of anti‐inflammatory factors, immune cell reprogramming, and regulating cell death pathway [707, 708, 709, 710, 711, 712, 713]. Especially, cfDNA and ROS will self‐amplify and potentiate each other during the inflammatory cascade, eventually resulting in severe tissue injury [714, 715, 716]. In a typical run, ROS induces cellular damage and releases cfDNA into the extracellular environment. Conversely, cfDNA acts as a DAMP, exacerbating inflammation by activating immune cells and stimulating the production of ROS, thus creating a vicious cycle that perpetuates inflammation and tissue damage [717, 718, 719]. Considerable advances have been achieved in constructing therapeutic agents that simultaneously eliminate pro‐inflammatory cfDNA and ROS to mediate inflammatory responses. For instance, Shi et al. [157] prepared a diselenide‐bridged MON‐PEI for the treatment of IBD. MON‐PEI exhibited high cfDNA binding affinity and ROS‐responsive degradation, and attenuated inflammation by alleviating cfDNA and ROS‐mediated inflammatory responses. Similarly, multifunctional TA‐Zn‐Gen NPs were employed for the treatment of sepsis [162]. It bound and scavenged pro‐inflammatory cfDNA, thereby inhibiting cfDNA‐induced TLR activation and the NF‐κB signaling pathway. The phenolic hydroxyl group in TA also chemically reacted with ROS to form more stable compounds, which can effectively attenuate ROS‐induced DNA damage and cell death. Besides, a biomimetic microrobot based on PLGA/PEI shell and Mg nucleus (Mg@PLGA@PEI) was proposed for IBD treatment, where the PEI shell adsorbed cfDNA, while hydrogen released by the Mg core significantly neutralized ROS, thus alleviating the inflammatory response [720]. Zhang et al. [721] created platinum‐doped positively charged carbon dots (Pt‐CDs) with strong ROS scavenging ability. During the pathological process of psoriasis, Pt‐CDs can effectively scavenge TNF‐α‐induced ROS and reduce the level of oxidative stress. It also significantly inhibited the cGAS‐STING pathway and restored cellular immune tolerance to cytoplasmic dsDNA, offering a comprehensive therapeutic option to target the multiple pathological mechanisms of inflammatory diseases.

Therapeutic agents can also be combined with immunomodulators to minimize immune activation and inflammatory response. Considering that H_2_O_2_, MPO, and neutrophils are upregulated in the inflammatory microenvironment, a nanomedicine named Lum/Ce6@PLGA, which responded to the inflammatory microenvironment, was proposed by loading chlorine E6 (Ce6) luminal (Lum) onto PLGA, which generated cytotoxic single oxygen under the action of the upregulated H_2_O_2_ and MPO, while killing pathogens and inducing the apoptosis of the excessive neutrophils in the inflamed site without disrupting immune homeostasis [722].

## 6 Therapeutic cfDNA‐Scavenging Strategies for Inflammatory Diseases

### 6.1 IBD

IBD is a chronic, persistent, and inflammatory intestinal disorder that primarily includes ulcerative colitis (UC) and Crohn's disease (CD) [723]. During intestinal inflammation, the innate immune system recognizes and responds to PAMPs and DAMPs, activating a series of pro‐inflammatory signaling pathways, which leads to the overproduction of inflammatory mediators. In turn, this exacerbates the inflammatory response in the intestine [724]. With a deeper understanding of the mechanisms of IBD and the complexity of biological signaling, novel strategies have emerged for the treatment of IBD [725]. Growing studies have shown that cfDNA released by damaged cells and intestinal microbes contributes to the extent and duration of inflammation by activating TLR9‐mediated signaling pathways in immune cells, playing an important role in the development of IBD [175]. Furthermore, oral administration is the most convenient route for drug administration in the clinical therapy of IBD, as a result of its preferred merits, such as non‐invasiveness, convenience, safety, and more importantly, direct action on the target sites [726, 727].

Jin et al. [728] synthesized a metal polyphenol network (Cur@Fe&TA) encapsulating Cur NPs and further explored its integrative mechanism in the treatment of IBD. Cur possessed significant antioxidant properties, which increased the expression of endogenous antioxidant enzymes (e.g., superoxide dismutase [SOD] and GSH) and protected intestinal cells from oxidative damage. By removing cfDNA, the release of inflammatory factors was down‐regulated, thus enhancing the therapeutic effect of Cur@Fe&TA against IBD. Using DNA‐based nanomaterials and available crystallographic data to activate immune cells, DNA origami NPs presenting CpG sequence were prepared in a rationally designed spatial pattern to activate TLR9 in RAW 264.7 macrophages [729]. When used as adjuvants in vaccines, NPs were rapidly taken up by antigen‐presenting cells (APCs) and subsequently bound to TLR9 in the nuclear endosome, triggering an intracellular signaling cascade for activation of immune cells. As an innovative therapeutic approach for IBD, PEI conjugated diselenide‐bridged mesoporous organosilica NPs (MON‐PEI) were formulated to attenuate the inflammatory response by targeting the scavenging of cfDNA and ROS in the colon [157]. It inhibited the cfDNA‐mediated TLR9 activation and lipopolysaccharide (LPS)‐mediated TLR4 activation in macrophages. Orally administered MON‐PEI accumulated preferentially in the inflamed colon, reduced the TLR9‐MyD88‐NF‐ĸB pathways mediated inflammation, thereby relieving the release of inflammatory mediators and alleviating symptoms [730]. Li et al. [731] designed Ac2‐26/OxbCD NPs (AON) based on ROS‐sensitive nanomedicines OxbCD for the loading of an anti‐inflammatory peptide Ac2‐26, and modification with specific DNA binding molecules. AON was capable of releasing the anti‐inflammatory peptide Ac2‐26 in an inflammatory environment triggered by excessive ROS, allowing Ac2‐26 to effectively target and repair the damaged intestinal tissues. It also scavenges ROS and cfDNA, reducing the inflammatory mediator in vivo, showing enhanced therapeutic efficacy on IBD. Zhou et al. [652] developed PICsome vesicles that bridged positively charged PEGylation peptides (PEG‐PG) with guanidinium side chains and loaded MTX for inflammation management. It underwent a charge reversal to dissociate the PICsome and release PEG‐PG and MTX in an inflammatory environment, enabling cfDNA‐ and MTX‐mediated immunosuppression in inflammatory diseases, including AID and IBD. Besides, an oral inflammatory colon targeted nanomedicine ICANs was proposed, utilizing MSN loaded with cerium oxide NPs (Ce NPs) to scavenge ROS and coated with polyacrylic acid to enhance their targeting. After oral administration, ICANs are directly localized at the inflammation sites and bound to the inflammatory epithelial cells to exert antioxidant effects [732]. It can also alleviate intestinal inflammation by removing cfDNA and ROS, decreasing intestinal inflammation caused by oxidative stress, and protecting intestinal epithelial cells. Recently, our group [733] developed PEI‐L/D‐tartaric acid (L/D‐TA) complexes templated PEI‐L/D‐TA@MON by mimicking biosilicification, which included four functional moieties. In this complex, PEI electrostatically attracted cfDNA, tetrathulfide bonds reductively reacted with ROS, silanol groups adsorbed LPS, and L/D‐TA enabled chiral recognition and inflammatory localization. During the synthesis, PEI‐L/D‐TA, with a large number of basic amines and carboxyl groups on its surface, was efficient at simultaneously serving as a catalyst, scaffold, and template to promote the hydrolysis of alkoxysilane and silica deposition. Following oral administration, PEI‐L‐TA@MON, whose preferential conformation stereoscopically matched with the mucosa, anchored onto the inflammatory intestine for lesion targeting, and eliminated LPS, ROS, and cfDNA, alleviating oxidative stress and inhibiting the inflammatory cascade to achieve IBD therapy. Herein, the reactants displayed multi‐functionality corresponding to both mesoporous formation and disease treatment.

### 6.2 RA

RA is a chronic autoimmune disease characterized by symmetrical inflammation and pain in the joints. In the pathogenesis of RA, the abnormal activation of immune cells plays a crucial role [734, 735]. Once TLRs are activated, they trigger the release of a large amount of pro‐inflammatory cytokines, such as TNF‐α, IL‐1β, and IL‐6 [736]. The treatment of RA mainly involves systemic administration and local injection through the joint cavity [737]. Based on a cfDNA degradation strategy, a bioinspired nanomedicine was developed by integrating DNase I into a 3th generation PLL dendritic polymer (G3K) nanogel for RA therapy [652]. It targeted inflamed joints, adsorbed, disassembled, and eliminated cfDNA, thereby radically mitigating the progression of RA. Liang et al. [473] used an exogenous DNase I to degrade excessive cfDNA to prevent the activation of the immune system, which effectively inhibited RA symptoms by down‐regulating local cfDNA and suppressing abnormal inflammation in DNase knockout mice. Specifically, a PEGylation polyimidazole artificial DNase was fabricated. By removing cfDNA, blocking the degradation of STING and the phosphorylation of IRF3 induced by cytoplasmic DNA, and inhibiting the expression of IFN‐β, it alleviated RA as well as Aicardi‐Goutières syndrome caused by DNase deficiency in rats.

The application of cationic polymers to remove cfDNA is another novel strategy for the treatment of RA. Peng et al. [529] developed a molecular scavenger for the treatment of RA by fabricating a series of cationic dendrimers with a polycaprolactone (PCL) linear backbone grafted with a high density of PAMAM. The polymer length can be precisely adjusted through controlled polymerization, while the charge density is regulated by branching. The authors investigated the combined efficacy of cationic dendrimers, both 384‐G2 and 384‐G3, in a rat model of collagen‐induced RA, and demonstrated that 384‐G2, as well as 384‐G3, scavenged cfDNA and inhibited TLRs recognition, thereby interfering with the cascading inflammatory response and showing good therapeutic efficacy. Wang et al. [158] developed an exosome therapeutic agent called MEX+cP by binding anti‐inflammatory M2 macrophage‐derived exosomes (MEX) to PLL and matrix metalloproteinase‐cleavable PEG. Positively charged PLL electrostatically interacts with cfDNA for the suppression of the immune response. In another case, a P‐Arg‐based cationic peptide chelate, PEG‐TK‐NP_Arg_, was synthesized for the treatment of RA [536]. A ROS‐sensitive TK fragment was introduced to responsively expose the cationic layer in the RA joint cavity. It actively eliminates cfDNA and inhibits the activation of TLR9. For immune regulation, a nanomedicine‐hydrogel composite named NiH is designed to allow active lymphatic drainage and accumulation [738]. NiH cleared the extracellular/internalized cfDNA and prolonged the release of the cGAS inhibitor RU.521(RU) to inhibit proinflammatory responses. It significantly down‐regulated the proportions of pro‐inflammatory T cells (including Th17 cells) and M1 macrophages, while increasing the proportions of Treg cells, M2 macrophages, and myeloid‐derived suppressor cells (MDSCs), thereby promoting immune homeostasis and tolerance. Moreover, Liang et al. [739] demonstrated cationic NPs (CNPs) formed by self‐assembly of PLGA‐b‐PDMA could remove cfDNA and inhibit the activation of primary synovial monocytes and fibroblast‐like synoviocytes. Compared with soluble polymers, CNPs showed a higher cfDNA scavenging capacity and more favorable biodistribution in inflamed joints. Liu et al. [740] synthesized a series of silica NPs brushes grafted with different contents of PDMA (SiNP@PDMA) to selectively remove cfDNA from inflamed joint cavities. It revealed that the core‐shell structure of SiNP@PDMA impacted its binding affinity with cfDNA, circulation time, and retention in the inflamed joint cavity, which ultimately affected the therapeutic efficacy. Furthermore, to alleviate the toxicity of PAMAM, PEG‐5000 was coupled to its surface to form PEG‐PAMAM with highly branched structure and reactive functional groups, which showed a strong binding and scavenging ability for cfDNA, effectively inhibited the up‐regulation of TLR9, and significantly inhibited the activation of the phosphorylated inhibitor of nuclear factor kappa B alpha (pIκBα)/p‐NF‐κB signaling pathway [741]. In addition, Chen et al. [742] employed dimethylamino groups to modify PDA to form charge reversal DPs that could bind negatively charged cfDNA for the treatment of RA. The DPs exhibited higher positive charge density, effectively inhibited LPS‐induced inflammatory response in vitro, and attenuated joint swelling, synovial hyperplasia, and cartilage destruction in RA rats.

### 6.3 Sepsis

Sepsis is a life‐threatening disease characterized by systemic inflammatory responses and multisystem organ failures [743]. Mechanistically, the immune‐inflammatory imbalance is usually initiated and driven by excessive TLRs, which recognize multiple inflammatory mediators, including LPS, ROS, and cfDNA [744]. Immunomodulatory therapy for sepsis has been a prominent research topic in recent years, aiming at improving the prognosis of sepsis by controlling excessive inflammatory response and restoring immune function [745, 746, 747]. Moreover, sepsis can result in serious complications. Huang et al. [460] identified the crucial role of free mtDNA in regulating alveolar macrophage activation during sepsis‐associated acute lung injury, and employed biocompatible hybrid protein nanomotors composed of recombinant DNase‐I and human serum albumin (HSA) targeting mtDNA clearance. Pulmonary delivery of DNase‐I/HSA nanomotors removed mtDNA from the lungs and enhanced sepsis survival by reducing lung inflammation and injury.

Employing PEI‐MSN as cfDNA scavengers, the cfDNA‐driven pro‐inflammatory effects via TLR‐MyD88‐NF‐κB pathway in peritoneal macrophages of mice were substantially suppressed for the treatment of severe sepsis [161]. On this basis, Liu et al. [162] grafted cationic PEI with zeolitic imidazolate framework‐8 (PEI‐g‐ZIF) in a simple one‐pot process. In a typical run, PEI‐g‐ZIF was internalized by endocytosis and blocked the recognition between CpG and TLR9, providing another approach for the treatment of sepsis. For a multiple anti‐inflammatory therapy, Li et al. [541] developed a novel nanomedicine named TMPP containing four components: TA, Mn, PMB, and PVP. It not only had antibacterial effects, but also removed a variety of inflammatory mediators, such as cfDNA and ROS, and inhibited excessive immune response. Coincidentally, Liu et al. [748] created multifunctional tannic acid‐Zn^2+^‐gentamicin NPs (TA‐Zn‐Gen NPs) that strongly bound cfDNA and scavenged ROS. As expected, it blocked cfDNA‐induced activation of TLRs and NF‐ĸB signaling, and reduced ROS‐induced DNA damage and cell death. Furthermore, a novel 2D, sheet‐like, cationic cfDNA scavenger called BP‐G1_AMP_ was fabricated using PAMAM (P‐G1) and antimicrobial peptides (AMPs) covered with black phosphorus (BP) nanosheets [749]. BP‐G1_AMP_ treated CLP rats by electrostatically adsorbing cfDNA and NETs. Particularly, Wu et al. [750] developed a self‐assembled multifunctional carbon monoxide nanogenerator (Nano CO) for the treatment of sepsis, which released CO in response to ROS, thereby scavenging bacteria and activating the heme oxygenase‐1/CO system. Nano CO demonstrated remarkable efficacy by binding to cfDNA, scavenging harmful reactive oxygen and nitrogen species (RONS) and bacteria, blocking macrophage activation, inhibiting focal death, and activating autophagy, thereby relieving the inflammatory storm in sepsis.

### 6.4 Periodontitis

The pathogenesis of periodontitis is closely related to dental plaque, which is a biofilm composed of bacteria, salivary components, and cellular debris that forms and proliferates on the tooth surface [751, 752]. Bacteria in plaque secrete toxins and metabolites such as LPS to stimulate the gum tissue and activate the host inflammatory response. In particular, TLRs play a vital role in the pathogenesis of periodontitis, which triggers an inflammatory cascade, and produce various inflammatory factors such as cytokines (e.g. IL‐1, IL‐6, and TNF‐α) and chemokines [753, 754]. Unfortunately, a prolonged inflammatory state leads to bone resorption and destruction of the periodontal supporting tissues, which may eventually result in loosening and loss of teeth.

Nanomedicines targeting inflammatory mediators and with antibacterial properties have been extensively studied in the treatment of periodontitis [755, 756]. For example, Chen et al. [757] engineered an injectable hydrogel named OCMC‐PAMAM‐G3 by grafting a cationic PAMAM‐G3 onto anoxidized carboxymethyl cellulose (OCMC) backbone. Once engaged in the periodontitis therapy task, OCMC‐PAMAM‐G3 was capable of capturing anionic PAMPs and DAMPs. It reduced the LPS and cfDNA levels, and attenuated inflammatory bone loss in a mouse model of ligation‐induced periodontitis by attenuating the LPS/cfDNA‐TLR4/9‐NF‐ĸB pathways. Xie et al. [758] prepared an amphiphilic polypropylene carbonate (PPC)‐PEI (PEPE) nanomedicine modified with gallic acid (GA) to treat periodontitis. First, PEI is bound to cfDNA through electrostatic interaction. Owing to the o‐phenyltriol structure and the double bond of carbon‐oxygen GA, the modification enabled the nanomedicine to target and bind to TLR2, where four hydrogen bonds were formed, including SER‐784(where two hydrogen bonds are generated), LYS‐783, and ASP‐642. The TLR4/MD2 complex also formed four hydrogen bonds with GA, including GLU‐593, TRP‐590, and LEU‐568 (where two hydrogen bonds are generated) [755]. Through these mechanisms, PEPE indirectly or directly reduced the amount of cfDNA and significantly inhibited TLRs signaling, providing a novel idea for the treatment of periodontitis. In another example, G3@SeHANs was synthesized by coating PAMAM‐G3 on the surface of the cationic dendrimer SeHANs [165], which was able to effectively scavenge cfDNA and had an antimicrobial effect on inhibiting excessive immune responses for the treatment of periodontitis. Besides, Yu et al. [759] created a multifunctional hydrogel (GPM) with potent antimicrobial, osteogenesis‐inducing, and ROS scavenging capabilities through a facile approach. By removing cfDNA, GPM suppressed the local inflammatory response, thereby reducing ROS generation. This process also helped to reduce oxidative stress, protect gingival tissues, and promote osteogenesis.

### 6.5 Psoriasis

Psoriasis is a common and recurring chronic inflammatory skin disease, with symptoms of reddish papules or plaques on the skin surface, covered with layers of silvery‐white scales [760, 761]. An abnormal response of the immune system drives the occurrence and development of psoriasis, where T cells and other immune cells are abnormally active, attacking keratinocytes, and leading to a rapid proliferation and inflammatory response in the skin [762, 763]. In recent years, several studies have elucidated the dual roles of cfDNA in psoriasis, acting as both a biomarker and a potential driver. Targeting the removal of cfDNA has become a prospective research direction for the treatment of psoriasis [764, 765, 766]. Liu et al. [532] used BGC‐MNs to remove cfDNA from skin and blood. By modifying a highly protonated bisguanidine structure, the binding capacity between chitosan and DNA was significantly enhanced. Furthermore, a series of PDMA‐modified silica NPs (CSP) with controllable PDMA length and particle size was fabricated to target cfDNA in the dermis for treating psoriasis. Among them, CSP with higher PDMA content showed potent inhibition on CpG recognition by TLR9 [356]. Özcan et al. [767] coupled MTX with gold NPs (GNP) for the treatment of psoriasis. It reduced the proliferation and differentiation of keratinocytes, and directly affected immune cells by blocking T cell proliferation, promoting apoptosis, and decreasing Th1 and Th17 cytokine production. Taking into account the dynamic interactions between the cGAS‐STING pathway and immune cells, a nano‐inhibitor composed of Pt‐doped positively charged carbon quantum dots (Pt‐CD) was designed for psoriasis [721]. Pt‐CD bound extracellular cfDNA to prevent the activation of the cGAS‐STING pathway in macrophages and reduce the subsequent secretion of pro‐inflammatory cytokines.

Recent studies have revealed that DNA can react with peptides such as LL37, which induces cellular activation. Liang et al. [768] designed cNPs with a hydrodynamic diameter of approximately 70 nm. These cNPs were prepared by self‐assembly of the diblock copolymer of PLGA and PDMA, PLGA‐b‐PDMA474, which has excellent cfDNA binding capacity through charge interactions. cNPs can competitively bind cfDNA from a DNA‐LL37 immune complex and form a more compact cNPs‐cfDNA complex, thus inhibiting DNA‐LL37‐induced cellular activation. cNPs also block TLR9 activation in primary mouse plasmacytoid dendritic cells (pDCs) and epidermal cells. There is also a strong interaction between cNPs and CpG, which dissociates the CpG‐LL37 complex to form a CpG‐polymer complex, effectively regulating the immune state in psoriasis.

### 6.6 cfDNA‐Targeting Therapies for Other Inflammatory Diseases

Other respiratory diseases impose substantial healthcare burdens on society. Chronic rhinosinusitis (CRS) is characterized by the symptom of a prolonged sensation of nasal obstruction that interferes with normal breathing [769, 770]. For CRS therapy, functional 2D TiS_2_ nanosheets (TLPG_A_) coated with linear polyglycerolamine (LPG_A_) were prepared and showed high biocompatibility and cfDNA removal efficiency [168]. Regarding COPD therapy, the use of cGAS and TLR9 gene knockout mice blocked the induction of NETs‐DNA in vivo. The use of superoxide dismutase mimics targeting mitochondria (mitoTEMPO) inhibited NETs generation, and the use of DNase I degraded NETs, which all reduced the infiltration of NETs and the expression of NF‐κB‐related inflammatory factors in the nasal smoke‐induced COPD mouse, alleviating the degree of airway inflammation and emphysema [95]. Besides, TLPG_A_ decreased cfDNA levels in the nasal secretions of ECRS patients, and effectively adsorbed and removed cfDNA from the inflammation site, thereby inhibiting cfDNA‐induced TLR9 activation and EET formation, and alleviating eosinophilic airway inflammation in experimental mice. The cfDNA scavenging strategy holds promise for the treatment of other respiratory diseases, including chronic rhinosinusitis with nasal polyps (CRSwNP) and bronchial asthma (BA). Tu et al. [771] synthesized cationic polyglycerol (PGA) with a range of hydroxyl/amine ratios and modified it on BP nanosheets. The results indicated that BP‐PGA50 had excellent cfDNA scavenging ability, good anti‐protein adsorption capacity, and low cytotoxicity. It inhibited the activation of TLR9, which in turn significantly reduced the formation of NETs [772].

Furthermore, obesity and overweight are noticeable public health problems and have become epidemics worldwide [773, 774]. Based on the elevated plasma cfDNA levels in obese people, Huang et al. [680] compounded PAMAM‐G3 with HSA to treat diet‐induced obese (DIO) mice. Since cfDNA‐activated macrophages are involved in adipose tissue inflammation and insulin resistance via TLR9, PAMAM‐G3 can consistently inhibit cfRNA and cfDNA to reduce complications in obesity [775]. After proposing cfDNA as an attractive target for the treatment of acetaminophen‐induced liver injury (AILI), Sun et al. [169] further integrated N‐acetylcysteine (NAC) with DNase I as a combined antioxidant and anti‐inflammatory strategy. Specifically, NAC attenuated clinically excessive acetaminophen liver injury by mitigating oxidative stress, while DNase I relieved inflammation by directly degrading cfDNA, providing a novel therapeutic option for AILI [335]. Besides, trauma‐induced death is associated with an excessive inflammatory response, and subsequent multi‐organ failure, trauma‐induced coagulopathy, and post‐traumatic infections. Xiao et al. [776] exploited a cationic hyperbranched polyaminoglycoside containing disulfide‐bridged HPT (ss‐HPT) for the clearance of cfDNA to attenuate post‐traumatic inflammation and hypercoagulable states. It displayed low cytotoxicity, potent cfDNA binding efficiency, and effectively inhibited the cfDNA‐induced inflammatory cascade response and hypercoagulable state with amelioration of multi‐organ damage. Zhou et al. employed PAMAM (G3‐8) as the initiator to initiate the ring‐opening polymerization of benzyl glutamate NCA (POBLG‐NCA) with alkyne groups in the side chains. Subsequently, they attached guanidine groups through click reactions to obtain a series of spherical cationic helical polypeptides (SPPs). Through electrostatic attraction, salt bridging, and the spatial limitation of rigid α‐helices, the efficient capture and stable clearance of cfDNA were achieved, which significantly inhibited the inflammatory response mediated by TLR9, reversed organ damage, and provided a brand‐new strategy for the treatment of SLE [373]. For the treatment of acute kidney injury, a manganous oxide nanoflower (Mn_3_O_4_) was proposed with superoxide SOD and catalase (CAT) activities [777]. It removes ROS produced and adsorbed cfDNA from the blood, alleviating acute kidney injury. In addition, Cheng et al. [481] employed MSN‐PEI to capture negatively charged cfDNA and modulated fibro‐adipogenic progenitors (FAP)‐mediated fibrotic process by decreasing the proportion of pro‐fibrotic Gal 3 macrophages through cfDNA‐mediated TLR7/9‐NF‐ĸB signaling pathway, which significantly improved the treatment of refractory orofacial muscle fibrosis.

Given the current research focus on using nanomedicines to intervene in cfDNA and cfDNA‐related inflammation signals for the treatment of inflammatory diseases, Table 3 provides a detailed overview of therapeutic cfDNA‐scavenging strategies for inflammatory disorders. In particular, nano‐therapeutics based on cfDNA‐intervention are presented to facilitate a comparison of their similarities and differences in the treatment of various inflammatory diseases, which include the design approaches of nanomedicines such as drug loading, nano‐carrier structure, and therapeutic targets or mechanisms, as well as animal models, delivery routes, and treatment efficacy. Notably, the physicochemical properties of nanomedicines, such as surface charge, geometric morphology, and surface modification, are the key factors in determining their therapeutic efficacy. Their payloads can exert synergistic therapeutic effects, achieving effective clearance of cfDNA, precise intervention in cfDNA‐related downstream signaling pathways, and effective regulation of the inflammatory microenvironment. When combined with different delivery routes, these enable efficient treatment of various diseases.

**TABLE 3:** Therapeutic cfDNA‐scavenging strategies for inflammatory diseases.

Diseases	Model Drug	TDDS	Therapeutic mechanisms	Animal Model	Effectiveness	Delivery routes	Ref.
IBD	MON‐PEI	Mesoporous silicon NPs with spheres	Reducing cfDNA and blocking the TLR 9 pathway.	DSS‐induced	Mouse survival percent, length of colon and body weight increase; stool consistency index, fecal bleeding index and DAI decrease.	p.o.	[157]
	AON	β‐cyclodextrin derived cationic NPs with spheres	Clear cfDNA, eliminate ROS.	DSS‐induced	Oxidative stress indicators MPO, MDA and H_2_O_2_ and levels of TNF‐α, IL‐1β and IFN‐γ decrease.	p.o.	[731]
	Cur@Fe&TA	Metal‐polyphenol network NPs with spheres	Clear cfDNA, anti‐inflammatory and antioxidant.	DSS‐induced	Colon length, body weight, OCC protein expression, and levels of SOD and GSH increase; levels of IL‐6 and IL‐1β decrease.	p.o.	[728]
	ICAN	Mesoporous silica NPs with spheres	Clear cfDNA, eliminate ROS.	DSS‐induced	Colonic damage score and levels of TNF‐α and IL‐6 decrease, body weight and colon length increase.	p.o.	[732]
	PEG‐PG‐MTX	Multi‐ion composite vesicles with spheres	Reducing cfDNA and blocking the TLR 9 pathway.	TNBS‐induced	DAI and Levels of inflammatory cytokines, iNOS and H_2_O_2_ decrease, lengths of mouse colons increase.	rect	[652]
RA	MEX+cP	Macrophage‐derived exosomes with cup‐shaped	Reducing cfDNA and blocking the TLR 9 pathway.	CIA‐induced	Levels of IL‐6, TNF‐α and IL‐1β, paw thickness and arthritis score decrease; synovial hyperplasia alleviate.	i.v.	[158]
	NiH	Nanomedicine in hydrogel	Reducing cfDNA and blocking the cGAS pathway.	CIA‐induced	Mouse paw thickness and levels of TNF‐α, IFN‐β and IL‐12 decrease; systemic immunosuppression enhace.	s.c.	[738]
	DNase I@G3KBPY	Bioinspired nanogels	Reducing cfDNA and blocking the TLR 9 pathway.	CIA‐induced	Extent of popliteal lymph node enlargement alleviate; Paw temperature decrease; inflammatory cell infiltrate alleviates.	i.v.	[835]
	SiNP@PDMA	Mesoporous silicon NPs	Reducing cfDNA and blocking the TLR pathway.	CIA‐induced	Swelling volume of hind paw decrease; bone and cartilage damage repair; levels of TNF‐α, IL‐1β and IL‐6 decrease.	i.v.	[740]
	PLGA‐b‐PDMA	PDMA‐derived cationic NPs	Reducing cfDNA and blocking the TLR 9 pathway.	CIA‐induced	Degree of joint swelling in rat decrease; bone loss repair; ankle, suprapatellar bursal effusion reduces Levels of TNF‐α, IFN‐α and IL‐6 decrease.	i.v.	[739]
	PEG‐PAMAM	PAMAM‐derived cationic NPs with spheres	Reducing cfDNA and blocking the TLR 9 pathway.	MIA‐induced	Number of apoptotic cells decrease; bone density increase; cartilage and bone damage alleviate.	i.v.	[741]
	DNase	Artificial DNase	Reducing cfDNA and blocking the TLR 9 /cGAS pathway.	CIA‐induced	Average hind paw swelling, inflammatory cell infiltration, cartilage erosion and bone destruction alleviate; clinical score of hind and forepaw in rats decrease.	i.v.	[473]
	DP	Cationic mussel‐derived polymer	Combine and purify cfDNA.	CIA‐induced	Levels of IL‐6 and TNF‐α and hind paw swelling in rats decrease; hyperplasia and cartilage destruction alleviate.	i.v.	[742]
	PEG‐TK‐NP	Polymeric macromolecular NPs	Reducing cfDNA and blocking the TLR 9 pathway.	CIA‐induced	Degree of hind paw swelling, inflammatory cell infiltration and cartilage and bone erosion alleviate.	i.v.	[536]
Sepsis	TMPP	PVP‐derived cationic NPs with spheres	Reducing cfDNA, ROS and blocking the TLR 9 pathway.	CLP‐induced	Survival, body weight, body temperature of rat increase; clinical score decreases; lung injury alleviates.	i.p.	[541]
	PEI‐g‐ZIF	PEI‐derived cationic NPs with spheres	Reducing cfDNA and blocking the TLR 9 pathway.	CLP‐induced	Survival of rat increase; body weight increase; clinical score decreases; inflammatory cell infiltration in the liver and heart alleviate.	i.p.	[162]
	BP‐G1AMP	Polymeric macromolecular NPs	Reducing cfDNA and NET.	CLP‐induced	Leukocyte infiltration and tissue damage alleviate; organ damage score decrease.	i.p.	[749]
	TA‐Zn‐Gen	Anionic NPs with spheres	Reducing cfDNA, ROS and blocking the TLR 9 pathway.	CLP‐induced	Bacterial population of the peritoneal cavity decrease; levels of TNF‐α and IL‐6 increase	i.p.	[748]
	MSN‐PEI	PEI‐derived cationic NPs with spheres	Reducing cfDNA and blocking the TLR 9 pathway.	CLP‐induced	Levels of TNF‐α and IL‐6 decrease; survival of rat's increase; liver and kidney injury score decrease.	i.p.	[160]
	DNase‐1/HSA NMs	DNAase‐human serum albumin hybrid protein nano‐engine	Reducing cf‐mtDNA and blocking the TLR 9 pathway.	LPS‐induced	Lung injury and inflammation alleviate; levels of TNF‐α, IL‐1β and IL‐6 decrease; degree of pulmonary tissue edema alleviate; survival rate increase.	inh	[460]
	Nano CO	Carbon monoxide NPs with spheres	Reducing cfDNA and ROS.	LPS‐induced	Survival rate increase; body weight increase; body temperature rise; levels of NO, TNF‐α and IL‐6 decrease.	i.p.	[750]
Periodontitis	GA‐PEPE	PEI‐derived cationic NPs with spheres	Reducing cfDNA and blocking the TLR pathway.	LPS‐induced	Levels of TNF‐α, IL‐6 and IL‐1β decrease.	s.c.	[758]
	GPM	Injectable nanocomposite hydrogel	Clear cfDNA, eliminate ROS.	P. gingivalis‐induced	Level of IL‐1β and OCN positive expression increase; alveolar bone regeneration capacity and bone healing capacity enhace.	s.c.	[759]
	OCMC‐PAMAM‐G3	Injectable cationic hydrogel	Reducing cfDNA and blocking the TLR 4/9 pathway.	Ligature‐induced	Alveolar bone loss alleviates; levels of TNF‐α, IL‐6 and MPO cell counts decrease.	s.c.	[757]
	G3@SeHANs	PAMAM‐derived cationic NPs	Reducing cfDNA and blocking the TLR 9 pathway.	Ligature‐induced	Levels of TNF‐α and IL‐6 and osteoblast activity decrease; bone loss alleviate.	s.c.	[165]
Psoriasis	BGC‐MNs	Biguanide chitosan microneedles	Combine and purify cfDNA.	IMQ‐induced	Erythema, scaling and thickness of dorsal skin in mouse mitigate; levels of TNF‐α, IL‐1β, IL‐6, IL‐17 and IL‐23 decrease.	t.d.	[532]
	cSPs	Cationic hairy NPs	Reducing cfDNA and blocking the TLR 9 pathway.	IMQ‐induced	Erythema, scaling and hardening of the skin alleviate; levels of TNF‐α, IL‐1β, IL‐6, IL‐17 and IL‐23 decrease; leucocyte infiltration reduce.	t.d.	[356]
	MTX‐GNP	Spherical gold NPs	Reducing cfDNA, inhibits T cell proliferation and induces apoptosis	IMQ‐induced	Ear thickness decrease; weight increase; echinoderm and papillomatosis score increase.	t.d.	[767]
	Pt‐CD	Platinum‐doped positive carbon dot NPs with spheres	Reducing cfDNA and blocking the cGAS pathway.	IMQ‐induced	Erythema, scaling and thickness of dorsal skin in mouse mitigate; levels of TNF‐α and IL‐6 increase.	t.d.	[721]
	CpG‐LL37	PDMA‐derived cationic NPs with spheres	Reducing cfDNA and blocking the TLR 9 pathway.	IMQ‐induced	Erythema, scaling and thickness of dorsal skin in mouse mitigate; levels of IL‐6, TNF‐α, IL‐17 and IL‐23 increase.	t.d.	[768]

Abbreviations: ALI, acute lung injury; CIA, collagen‐induced arthritis; CLP, cecal ligation and puncture; DAI, disease activity index; DSS, dextran sodium sulfate; GSH, glutathione; IL‐12, interleukin‐12; IL‐17, interleukin‐17; IL‐23, interleukin‐23; IFN‐α, interferon‐α; IFN‐β, interferon‐β; IMQ, lmiquimod; inh, inhalation; i.p., Intraperitoneal injection; i.v., intravenous injection; LPS, lipopolysaccharide; MDA, malondialdehyde; MPO, myeloperoxidase; mtDNA, mitochondrial DNA; OCC, overexpressed in colon carcinoma; OCN, osteocalcin; p.o., oral administration; rect, rectal administration; ROS, reactive oxygen species; s.c., subcutaneous injection; SOD, superoxide dismutase; t.d., transdermal patch; TDDS, targeted drug delivery system.

## 7 Conclusions and Perspectives

In recent years, the dual roles of cfDNA in inflammatory diseases as both a biomarker and a potential pathogenic driver have been increasingly recognized. Substantial foundational research efforts are critically required to standardize cfDNA testing protocols and establish unified analytical and reporting frameworks. Furthermore, cfDNA detection technologies have progressively integrated with multi‐omics analyses, including genomics, fragomics, and epigenetics. Until now, machine learning models and clinical epidemiology have been utilized in comprehensive evaluations to enhance the clinical utility of cfDNA in prognostic assessment and therapeutic efficacy monitoring.

Given the physical, biochemical, and immunological properties of cfDNA, therapeutic approaches capable of inhibiting its generation, scavenging existing cfDNA, and blocking its downstream inflammatory signaling pathways may mitigate organ damage in inflammatory diseases by modulating immune responses and suppressing pro‐inflammatory cascades. Upon evaluating such interventions, key considerations such as efficacy, safety, targeting specificity, and responsiveness should be comprehensively assessed and tailored to their intended clinical applications. From another perspective, the relationship between the physicochemical properties of cfDNA‐targeting agents and their innate immune responses requires a systematic exploration, particularly their regulatory effects on various cell types, signaling pathways, and inflammatory mediators. Translating these mechanistic insights into clinically actionable diagnostic and therapeutic strategies for inflammatory diseases remains an area requiring further exploration. Nanomedicine offers a promising approach for the intervention of cfDNA. The rational design of parameters, such as composition, particle size, surface charge, chemical modifications, formulation forms, and delivery routes, can optimize the therapeutic performance of the corresponding nanotherapeutics. Additionally, more precise and personalized strategies based on nanomedicine are expected to emerge, facilitating the targeted removal of cfDNA and improved disease management.

While recent reports have indicated that tumor gene profiling can be conducted through ctDNA testing in clinical practice to guide targeted therapy for patients with advanced gastrointestinal tumors [778], the clinical translation of cfDNA‐targeted therapy in inflammatory diseases still faces several challenges. First, biocompatibility and safety present fundamental challenges for cfDNA‐targeting nanotherapies. While the successful application of LNP‐mRNA vaccines highlights the potential of nanotherapy, most nanomedicines suffer from complex structures and preparation processing, leading to significant batch‐to‐batch variations that pose challenges for structural characterization and quality control. Moreover, most currently existing nanomedicines, including polymer‐based, lipid NP, and inorganic NP formulations, face the issue of nondegradability, risking long‐term accumulation in vivo. Especially, cytotoxicity associated with high surface charge density limits their therapeutic applications. Additionally, due to their microscopic size and complex structures, conventional characterization methods may not be effective. For these ends, optimal nanomedicine design still requires careful balancing of cfDNA‐scavenging efficiency against cytotoxicity through precise tuning of composition, particle size, charge density, and functional group modifications. Furthermore, advanced characterization techniques, including cryo‐electron microscopy (Cryo‐SEM), atomic force microscopy (AFM), and synchrotron radiation, should be integrated to comprehensively evaluate both the structural and functional properties. Second, species differences and individual heterogeneity lead to poor correlations between preclinical findings and clinical trials, complicating predictions about their efficacy and safety. In preclinical studies, the diversity of animal species, cell types, and complex in vivo environments makes data originating from different literatures incomparable. While organoid and ex vivo human samples can effectively reduce species differences, most nanomedicines still lack sufficient clinical validation. Especially for structurally complex nanomedicines, particular attention must be paid to architectural reproducibility and its correlation with clinical efficacy. Comprehensive characterization of in vivo dynamics and pharmacokinetic profiles of nanomedicines is essential for robust development and large‐scale production. However, the lack of unified regulatory standards for nanomedicines, combined with high R&D costs, extended development cycles, and the necessity to demonstrate their clinical advantages and commercial viability over conventional drugs, has increased investment concerns, significantly hindering the progress of nanomedicines.

## Author Contributions

Jiatong Li, Yumei Wang, and Xiaoxi Fan drafted and conceptualized the initial manuscript. Yumei Wang, Jiatong Li, Miao Xu, and Jiawen Li designed and organized figures and tables. Supervision and strategic direction were provided by Heran Li, Jianxiang Zhang, and Weifei Tan, who also contributed key ideas that shaped the manuscript. Heran Li and Jianxiang Zhang refined the manuscript. All authors have read and approved the review article.

## Conflicts of Interest

The authors declare no conflicts of interest.

## Data Availability

The authors have nothing to report.
